# Carbon Nanotube (CNT)-Based Biosensors

**DOI:** 10.3390/bios11120486

**Published:** 2021-11-29

**Authors:** David C. Ferrier, Kevin C. Honeychurch

**Affiliations:** 1 Institute of Bio-Sensing Technology, Frenchay Campus, University of the West of England, Bristol BS16 1QY, UK; david.ferrier@uwe.ac.uk; 2 Centre for Research in Biosciences, Frenchay Campus, Department of Applied Sciences, University of the West of England, Bristol BS16 1QY, UK

**Keywords:** CNT-based biosensors, bioanalytical applications, surface functionalization, electrochemical biosensing, analytical nanodevices

## Abstract

This review focuses on recent advances in the application of carbon nanotubes (CNTs) for the development of sensors and biosensors. The paper discusses various configurations of these devices, including their integration in analytical devices. Carbon nanotube-based sensors have been developed for a broad range of applications including electrochemical sensors for food safety, optical sensors for heavy metal detection, and field-effect devices for virus detection. However, as yet there are only a few examples of carbon nanotube-based sensors that have reached the marketplace. Challenges still hamper the real-world application of carbon nanotube-based sensors, primarily, the integration of carbon nanotube sensing elements into analytical devices and fabrication on an industrial scale.

## 1. Introduction

Carbon nanotubes (CNTs) have become one of the most widely utilised nanomaterials, with thousands of papers being published over the last two decades. There is a great variation of CNT forms and as this highly active field of research grows, further forms are being modelled and fabricated. Hence, there is a great deal of attention focused on CNTs in many fields. One field of particular interest is their application to the field of biosensors. Their unique structure and nanoscale dimensions mean that CNTs possess many properties that are of great benefit for chemical and biological sensing applications. These properties include a very large surface area to volume ratios and large aspect ratios, excellent electrical conductivities, a high chemical stability, and fluorescent properties. The application of CNTs in the field of biosensors has focused on electrochemical [1,2,3,4], optical [5,6,7], and field-effect devices [8,9]. To exploit the remarkable properties of this nanomaterial in biosensor development, CNTs need to be purified and functionalised with biorecognition elements. The design of the biosensing interface constitutes the key challenge in biosensor development. It needs to consider both the functionalisation and transduction steps. In order to achieve this, the immobilisation of the biosensing element must be optimised so that the analyte can be selectively recognised at the biosensor surface. Transduction must also be optimised so that small changes in the biorecognition element resulting from the presence of the target analyte can be detected rapidly and sensitively.

This paper will include an overview of CNT-based biosensors, but the main focus will be on the most notable advances in the field. Initially, the structure, form, and main synthesis methods of CNTs will be described, followed by the key strategies used for functionalising CNTs. Secondly, how CNTs can be used in the preparation of biosensors will be discussed. Finally, the technical and industrial challenges facing the integration of CNTs for application in biosensor systems will be discussed.

## 2. Structure and Form of Carbon Nanotubes

CNTs have a one-dimensional cylindrical shape with nanometre scale diameters and micrometre scale length. Iijima first reported multiwalled carbon nanotubes (MWCNTs) and their preparation based on results obtained in investigations of an arc evaporation method for the fabrication of C_60_ fullerenes in 1991 [10]. However, CNTs were previously described in 1952 by Radushkevich and Lukyanovich [11] and in 1976 by Oberlin et al. [12] who reported on the presence of single (or double) walled carbon nanotubes.

**Figure 1:**
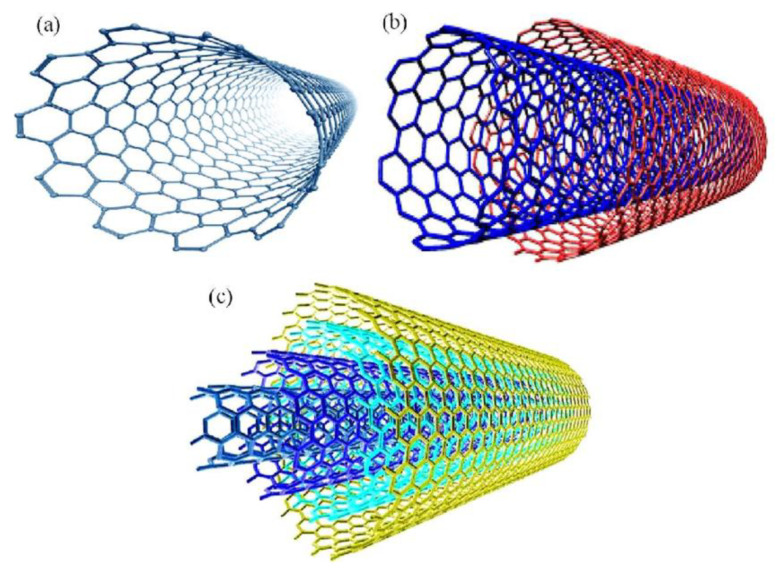
Structure of (**a**) a single-walled carbon nanotube (SWCNT), (**b**) a double-walled carbon nanotube (DWCNT), and (**c**) a multi-walled carbon nanotube (MWCNT). Reprinted with permission from Rafique, I. et al., 2016 [15]. Copyright 2016 Taylor & Francis.

Structurally, CNTs can be thought of as a rolled-up sheet of graphene. CNTs can be grouped into two main types, single-walled carbon nanotubes [13] (SWCNTs) and multi-walled carbon nanotubes [14] (MWCNTs), (Figure 1). The diameter of a MWCNT depends on the number of concentric nanotubes. The external diameter is generally less than 2 nm for SWCNTs. Various authors consider SWCNTs as a single large molecule, whereas MWCNTs are considered as a mesoscale graphite system with diameters ranging between 2 and 200 nm. MWCNTs can be divided into two different types that can be described by two different models. In the Russian Doll model, separate sheets of graphene are arranged in concentric cylinders, one inside of the other. In the alternative Parchment model, a single sheet of graphene is rolled in around itself, in a similar manner to a scroll of parchment or in a roulade cake.

With their morphology and properties similar to those of SWCNTs, double-walled carbon nanotubes (DWCNTs) can be viewed as a special class of CNTs. Notably, they are more chemically stable and as a result are more resistant to the relatively harsh chemical steps needed for functionalisation.

**Figure 2:**
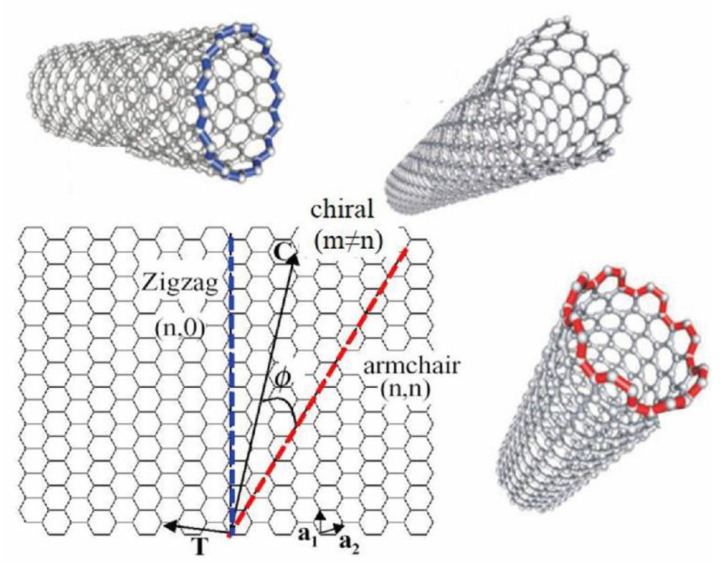
The construction of a carbon nanotube from a single graphene sheet. The chiral vector denotes different types of nanotubes [17].

The manner in which the graphene sheet is ‘rolled-up’ to make a single tube can have a bearing on the behaviour of the resulting CNT. For a SWCNT to be formed, two of the hexagon rings in the flat graphene lattice sheet need to be ‘rolled-up’ and overlapped. The direction, or vector, that this ‘rolling up’ follows across the graphene sheet is referred to as the chiral vector (Figure 2). Three different chiral vectors can be made, and these give rise to the three different possible CNT structures: zigzag, armchair, and chiral. The resulting structure formed is controlled by the two integer coefficients of the chiral vector, (n, m). If these values are equal, the armchair structure is formed. If the two coefficients are different, then the zig-zag form results. If m = 0 then the chiral form results. It is this direction of ‘rolling’ across the graphene sheet that governs the structure and properties of the resulting CNT [16]. The chiral dependence controls the electronic structure of the resulting CNTs and so governs the resulting semi-conductive or metallic-like properties exhibited by the CNT. However, experimentally it is difficult to control the chirality during the synthesis of CNTs and hence the resulting properties.

The physical and chemical properties exhibited by CNTs have been extensively recently reviewed [16]. CNTs have a very high melting point and a high tensile strength as each carbon atom is joined to three other carbon atoms by strong covalent bonds. This also leaves each carbon atom with a spare electron, which forms a sea of delocalised electrons within the tube, allowing for good electrical properties, and redox reactions with fast electron-transfer rates [18]. Importantly, CNTs exhibit good biocompatibility, allowing for integration with biological components, such as enzymes, DNA, and antibodies commonly employed in biosensor devices. These properties and their semi-conductive and metallic-like properties have led them to find varied applications in analytical and bioanalytical fields. However, questions have been raised about the potential toxicity [19,20,21] of nanomaterials and possible environmental issues [22].

## 3. Synthesis of Carbon Nanotubes

A number of reviews have been given on the synthesis of CNTs [23] and numerous differing approaches, such as top-down [24] or bottom-up [25] synthesis protocols have been reported. There are six commonly reported methods for synthesising CNTs:

arc discharge,
laser ablation,
chemical vapour deposition
plasma enhanced vapour deposition
liquid electrolysis, and
controlled flame environments.

The arc discharge method consists of applying a potential difference of around 20 V between two graphite electrodes held 1–3 mm apart in an inert atmosphere, typically argon. The resulting arc discharge evaporates graphite from the anode, which then condenses at the cathode, forming the CNTs. When pure graphite electrodes are used, MWCNTs are produced. The addition of a metal catalyst (such as iron, nickel, or cobalt) to the anode results in the formation of SWCNTs. The arc discharge method is incapable of controlling the purity of CNTs and further chemical steps are needed to obtain pure CNTs.

In 1995, the synthesis of SWCNTs by laser ablation was first reported [26]. In this method a target containing a mixture of graphite and a metal catalyst under a flow of inert gas, such as argon is exposed to a laser resulting in evaporation of graphite. Carbon nanotubes are then recovered from the resulting condensate [27,28,29]. However, this approach also leads to the formation of impure CNTs, again requiring further chemical treatment.

Chemical vapour deposition (CVD) [30,31] has been shown to be a very promising approach for the synthesis of CNTs. This method is based on the decomposition of a carbon containing gas such as methane, ethylene, acetylene, and carbon monoxide [32] at the surface of a heated metal catalyst. Both SWCNTs and MWCNTs can be synthesised using the CVD method. MWCNTs with an inner diameter of about 1–3 nm and an outer diameter varying from 2 to 20 nm can be readily made; however, the synthesis of SWCNTs has been found to be difficult to control.

Improvements to the CVD process have been made by the incorporation of an additional plasma process: referred to as plasma enhanced CVD (PECVD) [33,34,35,36,37,38,39]. In this approach, the activation of the gas is carried out using electron impact instead of via thermal energy, allowing for the possibility of CNT synthesis at lower temperatures. This has made the PECVD a more attractive process, as it can be applied for the synthesis of CNTs on a greater range of potential substrates (such as glass or silicon) as it does not damage them. PECVD is used to synthesise vertically aligned CNTs at temperatures ranging from 400 to 650 °C [40,41,42,43,44,45,46,47,48].

The mechanism for the liquid electrolysis method is similar to CVD. Here, metal ions are reduced to their native metal form at a cathode and act as nucleation points for the growth of CNTs [49]. The process is reported as a green approach, as metal carbonates such Li_2_CO_3_ can be used. This allows for the overall reaction to result in the consumption of the greenhouse gas, carbon dioxide, to give CNTs.

Fullerenes and CNTs are formed in everyday flames and can be found in the resulting soot [50]. However, the conditions in these naturally occurring flames are generally uncontrolled, and the resulting fullerenes and CNTs formed can be highly irregular in size and quality. More controlled flame-based methods have been reported [51].

## 4. Functionalisation of Carbon Nanotubes

The functionalisation of CNTs has received a great deal of interest as their carbon chemistry allows for a range of different modifications to be made. A range of areas has been explored, such as minimising aggregation, improvement of their aqueous solubility and their biocompatibility. Carbon nanotubes have a large surface area, allowing for the addition of a large number of functional groups. However, in practice, the bundling effect, as well as the increase in the number of walls, decreases the surface area available [52]. Aggregation can be a problem with the application of CNTs and three methods to minimise this have been reported. The first is based on covalent modification, grafting functional groups to the sp^2^ carbon atoms of the CNTs [53,54]; the second method is based on non-covalent modification, by the adsorption of functional groups by hydrophobic chemical structures [55]; and the third method is completed by forming defect groups of functionalisation on the ends of the CNTs and its sidewalls [56].

The most commonly used approaches for the functionalisation of CNTs are based on chemical, physical, or electrochemical processes. For the development of biosensors [57,58,59,60,61,62,63,64], Carbon nanotube functionalisation has been undertaken for three reasons: (i) to modify the substrate surface with ordered, anchored CNTs, (ii) the addition of biosensing elements to the CNTs and (iii) for the modification of their optical and electronic properties required for the desired application.

### 4.1. Chemical Functionalisation

A number of different methods have been developed to both alter and increase the functionality of CNTs. The most commonly reported is the application of acid-based oxidisation for the introduction of oxygen containing functional groups, such as carboxyl, acyl, and hydroxyl groups. This process is generally undertaken using nitric or nitric/sulphuric acid mixtures and is effective for increasing oxygen containing functional groups to both the ends and the sidewalls of the CNTs [65,66]. However, the effect of oxidative treatment can vary greatly. Nevertheless, oxidative treatment has been found to increase the hydrophilic character of CNTs but control of the corresponding deterioration of the electrical, optical, and electrochemical properties of CNTs is often difficult. Oxidative treatment can also result in the introduction of defect sites, which can lead to undesired chemical and physical properties [67].

**Figure 3:**
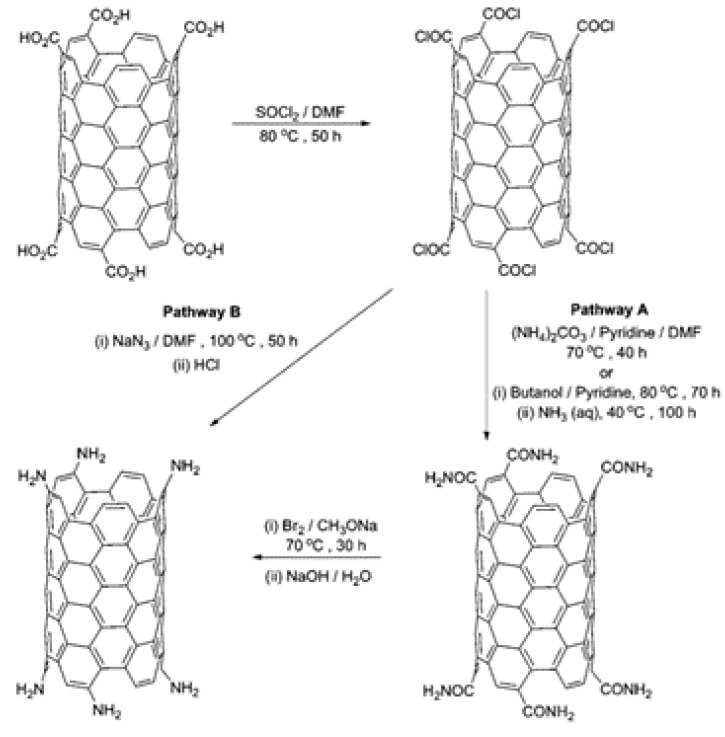
Preparation of amino-functionalised SWCNTs via the Hofmann rearrangement of carboxylic acid amide (pathway A) and via the Curtius reaction of carboxylic acid chloride with sodium azide (pathway B). Reprinted with permission from Karousis, N. et al., 2010 [70]. Copyright 2010 American Chemical Society.

The addition of these oxygen containing functional groups allows for the further modification of the CNTs. These can be used as precursors for addition or conversion to give new functional groups. Amino groups have been obtained using two methods [68,69]: (i) the Hofmann rearrangement of the corresponding amides and (ii) the Curtius reaction of an acyl chloride with a suitable azide (Figure 3).

Most of the strategies reported in the literature indicate the difficulty in integrating an ordered molecular structure onto the CNT surface. This difficulty is often due to the incompatibility between the functionality of the chemical structure desired and the experimental conditions required for CNT functionalisation. The use of the click chemistry concept offers some interesting possibilities for CNT functionalisation for different applications [70,71,72,73,74,75,76,77,78,79,80,81,82]. The best-known reaction of click chemistry is the Cu (I)-catalysed azide-alkyne 1, 3-dipolar cycloaddition (CuAAc), which was successfully used in the preparation of biosensors. This method may be achieved in mild experimental conditions and coupled to the diazonium method. The major advantage of this method is the control of the molecular architecture of the electrode surface [83].

Chemical functionalisation of CNTs is still challenging due to the difficulty of obtaining a well-ordered molecular architecture of the electrode surface in a reproducible way and under mild experimental conditions. A well-organised molecular architecture, based on supramolecular chemistry, can be obtained by combining various methods including the diazotation process, click chemistry, and the incorporation of metal nanoparticles. However, it has been shown to be possible to use the approach of diazonium fabrication to form multiple functionalised electrode arrays [84,85,86]. It is worth noting that the effect of the functionalisation of both electronic and optical properties is different depending on the nature of the CNTs (SWCNTs or MWCNTs). For instance, a compromise must be found between increasing the functionalisation coverage and decreasing SWCNTs’ conductivity. Covalent functionalisation of SWCNTs may be replaced by non-covalent functionalisation in order to minimise the change in network resistivity of CNTs. The resistivity of MWCNTs is generally less sensitive to the effects of the functionalisation processes; the conductivity is still maintained through the inner walls even if the MWCNTs are modified.

Strong acid-treatments used in chemical processes for CNTs modifications were found to not only be effective in introducing oxygen-containing functional groups [87,88,89,90,91,92,93], but also to effectively remove metallic catalysts from CNTs [94,95]. However, the acid oxidation of CNTs is limited by the time required for the process, and this is often considered inappropriate in industrial applications.

### 4.2. Physical Functionalisation

Alternative approaches have been explored for functionalising CNTs by introducing oxygen-containing groups on the end and sidewall of CNTs. Among these approaches, plasma treatment has been explored to graft various functional groups such as carboxylic, hydroxyl, or amine groups. For this purpose, a series of plasma gases could be used, such as oxygen, air, a mixture of hydrogen and nitrogen, or carbon dioxide. Plasma processes have also demonstrated an effective way of increasing the hydrophilic character of CNTs, thus facilitating the immobilisation of biorecognition elements for the fabrication of biosensors.

In the case of an aligned configuration of CNTs, microwave plasma treatment using carbon dioxide or nitrogen/hydrogen not only permitted the functionalisation of the CNTs but also avoided the aggregation phenomena, thus retaining the alignment structure of the electrode surface [93]. The atmospheric plasma treatment was demonstrated to be more appropriate for the functionalisation of CNTs with an aligned configuration compared to the classical oxidative treatment in a solution.

### 4.3. Non-Covalent Functionalisation

Non-covalent functionalisation is very useful since it offers the possibility to modify CNTs without damaging the sp^2^ structure, which may cause a significant change in both the conductivity and optical properties of CNTs. The exploitation of π–π interactions for stabilising the dispersion of CNTs in suspensions has been widely reported. For instance, the current monovalent functionalisation of CNTs includes π–π stacking of aromatic groups (e.g., pyrene) and sidewalls of CNTs. The 1-pyrene-butanoic acid succinimidyl ester has been used to covalently bind biomolecules to CNTs [56]. The non-covalent functionalisation of SWCNTs could be considered to be the most suitable for preparing biosensors based on luminophor systems [96,97] and has been widely applied to DNA sensors [98,99,100,101,102].

### 4.4. Electrochemical Functionalisation

Electrochemistry may offer the possibility to functionalise CNTs under mild reaction conditions whilst avoiding undesired and uncontrolled reactions. Diazonium salt reactions provide interesting approaches for functionalising the end-tip and sidewall of CNTs [103,104,105,106,107,108,109,110,111,112,113,114,115,116,117]. Interesting results have been obtained with diazonium salts for functionalising CNTs; however, obtaining monolayers with a well-controlled architecture remains a challenge due to the spontaneous polymerisation process of radical species, which are formed from the decomposing diazonium salts. The diazonium salt method can cause the CNTs to anchor on an electrode surface [104]. It has been demonstrated that a well-organised assembly of CNTs is obtained, which provided a simple approach for modifying electrode surfaces with CNTs. The advantage of aryl diazonium salt derivatives is that they can be applied to a variety of surfaces, such as CNTs decorated with metal nanoparticles.

The modification of electrode surfaces with aryl diazonium salts is obtained by the reductive adsorption of one electron from the radical aryl diazonium. The electrochemical regeneration of the radical species is achieved at a working potential value of about 0 V versus the saturated calomel electrode (SCE), either in acetonitrile or in aqueous acid solutions with a pH of below 2 [118,119]. A stable covalent C–C bond with the carbon electrode is formed. However, the formation of the radical aryl diazonium often leads to multilayer structures instead of a well-ordered monolayer. This is the most significant drawback to electrochemical functionalisation using diazonium salts.

The side reactions in which radical aryl diazonium is involved are not only dependent on applied potentials or electrolysis time but are also dependent on the nature of radical group R and the reactivity of the electrode surface. Depending on the nature of R, the radical aryl diazonium may undergo a polymerisation process involving attacks on the ortho-position. An appropriate control of the charge passed during the electrochemical functionalisation may be used to avoid the formation of multilayers [120]. The functionalisation of CNTs by aryl diazonium was suggested as a way to facilitate their solubility in organic and aqueous solutions. Tour et al. [121] reported that by using a variety of diazonium salts, modification of the sidewalls of SWCNTs occurred. The functionalisation was studied, and the chemical structure of carbon surface was monitored by a variety of spectroscopic techniques. It is worth noting that the Raman D-band measurements indicated that multilayers could easily be grown on the sidewalls of CNTs. The intensity of the D-band is often used as an indicator to access the number of carbon atoms of SWCNTs transformed from sp^2^ to sp^3^. The authors demonstrated that the intensity of the D-band remained constant after the functionalisation process, proving that aryl diazonium rings were not grafted to the sidewalls of CNTs but to the aromatic itself.

On the whole, various studies on the electro-grafting of molecules and biomolecules have been reviewed by using several precursors including amines, carboxylic groups, alcohols, vinylics, and diazonium salts. Generally, any precursor can be grafted onto the surface of the CNT, as long as it is able to generate radical species. The in situ generation of aryl radical species was found to be very useful for the functionalisation of CNTs, but this process needs to be improved to better control the formation of multilayers [122], which often leads to a significant passivation of the electrode surface. The nature of precursors is fundamental for minimising the polymerisation processes and optimising the homogeneity of the structure of the surface. It is obvious that a better understanding of both the chemistry and electrochemistry of precursors will provide valuable knowledge in the field of electrochemical functionalisation. This will lead on to more reliable control to obtain a homogeneous and monolayer structure.

### 4.5. CNT/Nanoparticle Hybrid Materials

Carbon nanotubes may be used as appropriate materials for the incorporation of noble metal nanoparticles (NPs) [69,123,124,125]. Since the first report dealing with noble metal NP/CNT nanohybrid materials, a series of manuscripts have focused on approaches for assembling NPs on CNTs. Some of these approaches [126,127,128,129] are based on layer-by-layer methods by exploiting positively charged gold nanoparticles (AuNPs). These are able to be anchored on functionalised CNTs. Other approaches lead to self-assembled anchoring of CNTs on the electrode surfaces by using AuNPs [130,131,132,133].

Many methods to prepare nanostructured hybrid material-based AuNP-CNT systems have been reported, each method providing specific properties in terms of size and density of the NPs. These methods include electrochemical deposition, chemical deposition, interaction of NPs with functionalised CNTs and physical methods.

From an analytical point of view, the combination of CNTs and metal NPs leads to a synergistic effect in terms of sensitivity and electrical connection. The functionalisation of AuNPs integrated into CNTs leads to a high surface-to-volume ratio, which is suitable for the preparation of better performing biosensors. Furthermore, surfaces with CNTs/AuNPs based on the use of host-guest supramolecular interactions constitute an effective way for immobilising enzymes on AuNPs [134]. The preparation of nanostructured electrode surfaces by electropolymerising polyfunctionalised AuNPs with 2-mercaptoethanesulfonic acid, 1-adamantanethiol and p-aminothiophenol has been demonstrated.

## 5. Biosensors Based on Carbon Nanotubes

Carbon nanotubes have been applied to a wide variety of different types of biosensors, exploiting different aspects of their advantageous physical and chemical properties. These biosensors can be categorised according to the type of transduction mechanism, the most common classifications being electrochemical, optical, and field-effect-based sensors.

### 5.1. Electrochemical CNT Biosensors

Electrochemical sensors represent the oldest category of biosensors, dating back to the Clark electrode in 1962 [135]. Electrochemical biosensors remain highly common today, as a result of their relative simplicity, low-cost, and portability. The high surface areas of CNTs and their excellent electrical properties mean that CNTs can be of great use in improving the performance of electrochemical sensors. They have been used to improve the immobilisation of biological recognition elements, increase the active surface areas of electrodes, and improve electron transfer, all of which can increase the sensitivity and lower the limit of detection of electrochemical sensors [136,137,138].

#### 5.1.1. Electrochemical Enzyme Sensors

Electrochemical enzyme sensors are perhaps the most prevalent type of biosensor, mainly due to the predominance of glucose sensing. CNT-based enzyme sensors have been developed for a range of potential applications, from glucose sensing [139,140] to the detection of the ripening of fruits [141] and the spoilage of fish [142].

Perhaps the simplest method for immobilising enzymes on CNTs at the electrode surfaces is by adsorption. The CNTs can be deposited onto the electrodes via methods such as drop-casting [143] or by direct synthesis of the CNTs onto the electrode surface [144]. The enzymes can be adsorbed to the CNTs in solution prior to deposition or directly onto CNT-functionalised electrodes. Alternatively, the enzymes can be covalently bonded to functionalised CNTs. A common example is the binding of enzymes to carboxylated CNTs via carbodiimide linkages [141,145].

More complex approaches include dispersing CNTs in ionic liquids [139] or in polymers—such as Nafion [146], chitosan [147] or polymer mediators [142]—before drop-casting these composite mixtures onto electrodes. Enzymes can then be bound to these films by methods such as adsorption [142], covalent bonding (such as with glutaraldehyde cross-linkers [146,147]), or ionic attractions [139]. There are also examples of groups who have drop-cast layers of CNTs, deposited redox-active polymer layers on top and covalently bonded enzymes to the polymer [148]. It is also common to form composites of CNTs with other nanoparticles to enhance the electrical characteristics of electrodes. Researchers have investigated enzyme sensors based on electrodes functionalised with composites of CNTs and nanomaterials such as gold [147] and platinum [140] nanoparticles and graphene [143].

Whilst the majority of the reported CNT-based enzyme biosensors are intended to detect the substrate of a given enzyme, some have been developed which detect analytes that act as inhibitors to the catalytic activity of enzymes. One such example is the biosensor for metal ions developed by Moyo et al. This group adsorbed horseradish peroxidase (HRP) onto a composite of MWCNTs and maize tassel. The presence of metal ions will inhibit the reduction of peroxide by HRP, allowing for the amperometric detection of lead and copper ions at μg/L concentrations [149].

A majority of reported CNT-based enzyme sensors utilise CNTs that are randomly distributed upon an electrode surface or within a polymer film. The popularity of this approach is primarily down to the ease with which this can be carried out. However, several examples of enzyme sensors based on vertically aligned CNTs (sometimes referred to as CNT ‘forest’ electrodes) have been reported. This represents an attractive approach as it can more fully exploit the favourable characteristics of CNTs to prepare sensitive and selective biosensors. These arrays of vertically aligned CNTs can be created either by the direct synthesis of CNTs (such as via CVD techniques [144,150]) or by the deposition of CNT dispersions onto substrates functionalised with Fe^3+^-precipitated hydroxides [151]. Enzymes can then be immobilised onto the ends of these vertically aligned CNTs either by adsorption [144] or through covalent bonding, usually via a carboxyl-functionalised end group of the CNT [150,151].

It is worth mentioning that CNTs have been investigated as a means of achieving direct electron transfer (DET) between redox enzymes and electrode surfaces. This is a difficult technical challenge due to the fact that the redox centers of enzymes are typically located inside insulated protein shells. DET has been successfully demonstrated using functionalised SWCNTs, by making use of their nanoscale dimensions and conductive properties to form ‘molecular wires’ between electrode surfaces and the redox cores of enzymes [152,153]. However, at the time of writing, there have been few reported examples of CNT-based enzyme biosensors exploiting DET. The electrochemical enzyme sensors discussed in this section are summarised in Table 1.

#### 5.1.2. Electrochemical Immunosensors

**Figure 4:**
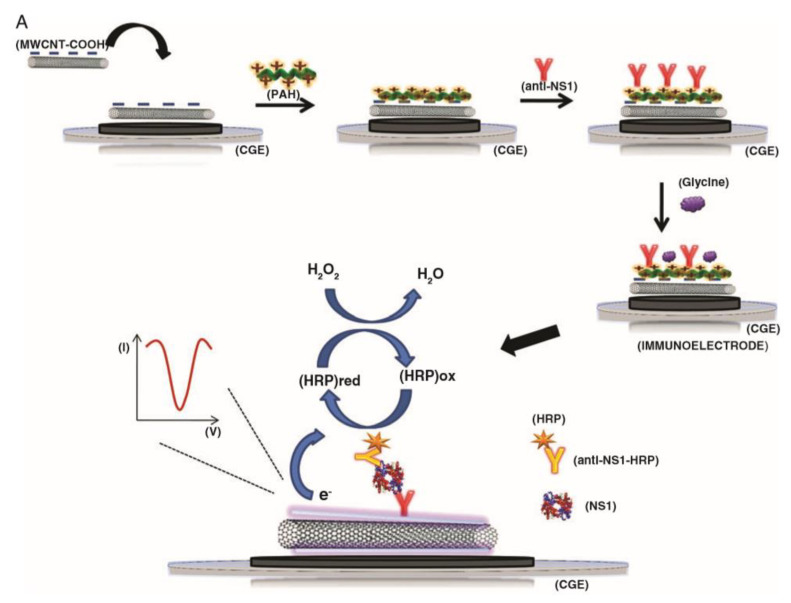
Schematic representation of the assembly and sensing mechanism of a sandwich type CNT-immunosensor. CNTs are immobilised on an electrode and antibodies are immobilised on top (in this instance via a poly(allylamine) layer). The analyte binds to the antibodies and then horseradish peroxidase (HRP)-labelled antibodies are introduced which will bind to the immobilised analyte. The presence on the analyte is then detected via the electrochemical signal from the HRP. Reprinted with permission from Silva, M.M.S. et al., 2014 [156]. Copyright 2014 Society of Chemical Industry.

Immunosensors are devices that exploit the binding interactions between antibodies and antigens. Either the antibody or the corresponding antigen can be immobilised at a sensor surface, and the binding of the corresponding member of the pair, forming a stable complex, can be detected via a variety of different approaches. Antibodies are widely used in biosensing applications because of the highly specific nature of their bonding with their corresponding antigens [154], and there are many examples of CNT-immunosensors. A common approach in CNT-immunosensors is the so-called sandwich assay. In this method, the electrode is functionalised with CNTs, antibodies are immobilised at the electrode—either directly to the CNTs or to another binding element at the electrode surface—and the binding of the analyte proteins are detected by the introduction of secondary antibodies functionalised with reporter molecules. The presence of these reporter molecules at the electrode surface, as they bind to the immobilised analyte, will result in a change in the electrical signal. This approach has been employed by the group of Dutra; they have immobilised CNTs at the surface of glassy carbon electrodes using polymer composites consisting of either polyethyleneimine or poly(allylamine) as the supporting polymer, and immobilised antibodies either via covalent bonding directly to the CNTs or via ionic bonding to the polymer. The binding of HRP labelled antibodies in the presence of the analyte molecules results in a change of current as the enzyme reduces peroxide (Figure 4). In this manner, this group has demonstrated sensors for the cardiac biomarker Troponin T to a detection limit of 0.033 ng/mL [155] and the Dengue virus NS1 protein to a detection limit of 35 ng/mL [156].

Riberi et al. [157] employed a similar approach in which MWCNTs are immobilised in a composite with polyethyleneimine and on top of which AuNPs are deposited by drop-casting. The antibodies are then adsorbed onto the AuNPs and a competitive assay is performed using HRP-labelled target molecules. In this manner, this group demonstrated an amperometric sensor for the detection of zearalenone (ZEA), a mycotoxin in maize, with a detection limit of 0.15 pg/mL.

Immunosensors based on vertically aligned CNTs have also been demonstrated. The Rusling group formed SWCNT forests by immersing graphite electrodes functionalised with a layer of Nafion and Fe(OH)_x_ into SWCNT dispersions [158]. Antibodies are then covalently bound to the ends of the CNTs and a sandwich assay is performed using HRP labelled antibodies. In this manner, this group developed an amperometric immunosensor for matrix metalloproeinase-3 (MMP-3), a biomarker associated with squamous cell carcinoma of the head and neck, with a detection limit of 4 pg/mL. The same group employed a similar approach for the detection of the oral cancer biomarker interleukin 6 (IL-6) protein and reported a detection limit of 0.5 pg/mL [159].

These amperometric approaches represent the most common type of electrochemical measurement in CNT-immunosensors. However, simplicity is an attractive quality in any sensor, and as such it is often desirable to remove the need for labelled reporter molecules (such as the HRP-labelled antibodies in the above examples) so as to reduce the number of steps and reagents involved in any given test. Immunosensors employing measurement techniques such as electrochemical impedance spectroscopy (EIS) do not require such label molecules. Arkan et al. [160] demonstrated such a label-free CNT-based immunosensor for the detection of human epidermal growth factor receptor 2 (HER2), a biomarker for breast cancer. By electroforming AuNPs on immobilised MWCNTs and binding antibodies to these nanoparticles via thiol linkers, this group developed a sensor in which the charge transfer resistance increases linearly with the binding of the analyte to the immobilised antibody. In this manner, they demonstrated the detection of HER2 in serum samples from breast cancer patients. Thapa et al. [161] used a similar approach to develop a sensor for the detection of the pancreatic cancer biomarker protein, CA19-9. They adsorbed MWCNTs onto layer of polyethyleneimine deposited on a gold electrode. The antibodies are covalently bonded to the CNTs via a carbodiimide linkage. The binding of the analyte to the immobilised antibody results in changes to the capacitance at the sensor surface, which can be measured by EIS.

Another method involves the use of redox probes such as ferricyanide. Kalyani et al. [162] developed a CNT-based immunosensor for the CA19-9 protein, a biomarker for endometriosis, utilising such a redox probe. A composite of MWCNT, magnetite (Fe_3_O_4_) nanoparticles and chitosan are deposited on a glassy carbon electrode and the relevant antibody bound to the chitosan via a glutaraldehyde linker. The binding of the analyte to the sensor surface impedes the electron transfer of the redox probe, resulting in a current drop. Using this approach and square wave voltammetry a detection limit of 0.163 pg/mL was achieved.

It is also possible to exploit the geometrical, rather than the electrical, properties of CNTs for immunosensing applications. Wan et al. [163] demonstrated an amperometric biosensor in which carboxylated MWCNTs are covalently bonded to the label antibodies in a sandwich assay of the type described previously. The high surface area of the CNT allows for multiple copies of HRP to be bonded to it, far more than can be bonded to a single antibody. This significantly increases the number of copies of HRP that are immobilised at the sensor surface as the result of a single analyte binding event, leading to a correspondingly greater response. This approach enabled this group to demonstrate the detection of the cancer biomarkers prostate specific antigen (PSA) and Interleukin 8 (IL-8) with detection limits of 5 and 8 pg/mL, respectively. The electrochemical immunosensors discussed in this section are summarised in Table 2.

#### 5.1.3. Electrochemical DNA Sensors

DNA presents an attractive biorecognition element for sensors because of its strong and highly specific hybridisation. DNA sensors have a wide range of potential applications including medicine and healthcare, food safety and counterterrorism [164].

A common method of immobilising nucleic acids onto CNT-functionalised electrodes is by covalent binding of the amine terminus of the nucleic acids. Chen et al. [165] fabricated a CNT-based DNA sensor by drop-casting a dispersion of copper oxide nanowires and carboxylated SWCNTs onto glassy carbon electrodes and covalently binding an amine-terminated DNA capture probe via a carbodiimide linkage. The selective hybridisation of the analyte DNA sequence is then detected by the use of adriamycin as an electrochemical indicator as it binds selectively to the double stranded DNA (dsDNA). Through the use of differential pulse voltammetry (DPV) this group were able to demonstrate the detection of the anthrax lethal factor to a limit of 3.5 fM. Ghera et al. [166] used the same method of covalently binding oligonucleotides to carboxylated MWCNTs, deposited onto indium tin oxide (ITO) electrode via electrophoresis. By using EIS to measure the change to the interfacial charge resistance caused by the hybridisation of the analyte sequence, this group demonstrated a sensor capable of detecting a DNA sequence specific to chronic myelogenous leukaemia in the femtomolar range.

It is also possible to immobilise nucleic acids at CNT-functionalised electrodes using gold/thiol interactions. Liu et al. [167] drop-cast a composite of MWCNTs and tungsten disulphide onto glassy carbon electrodes and formed gold nanoparticles on the surface of the composite via electroformation. Thiolated oligonucleotide capture probes are then bonded to the gold nanoparticles and the hybridisation of the analyte sequence detected via a HRP labelled probe. In this manner this group demonstrated the successful detection of hepatitis B virus genomic DNA to a limit of 2.5 fM using DPV.

Chen et al. [168] functionalised amine-modified MWCNTs with gold nanocages. These were drop-cast onto carbon screen-printed electrodes and thiol-modified RNA probes bound to the gold nanocages. In this manner this group demonstrated the detection of long non-coding RNAs (lncRNAs)—specifically metastasis-associated lung adenocarcinoma transcript 1 (MALAT1), a biomarker for non-small cell lung cancer—using differential pulse voltammetry to a detection limit of 42.8 fM.

Sabahi et al. [169] electroformed gold dendritic nanostructures on drop-cast SWCNTs. Thiolated PNA probes, a synthetic DNA analogue, are then bound to the gold nanostructures and DPV used to detect the hybridisation of cadmium ion functionalised RNA analyte sequences. This group demonstrated the detection of microRNA 21, a potential biomarker for many forms of cancer [170], to a limit of 0.01 fM.

DNA sequences can also be immobilised on CNTs via non-covalent binding. Ozkan-Ariksoysal et al. [98] bound DNA capture probes to MWCNTs in solution via π–π interactions. The terminal amine groups of the DNA are then covalently bonded to the carboxyl groups of a pencil graphite electrode immobilising both the DNA and the CNT at the electrode surface. The hybridisation of the analyte sequence is then detected via changes to the guanine oxidation signal. This approach has allowed the detection of a DNA sequence specific to *E. coli* to a detection limit of 16.7 nM.

Vertically aligned CNT forest electrodes have also been applied to DNA sensors. Li et al. [171] fabricated arrays of vertically aligned MWCNTs by PECVD from nickel catalyst films on a silicon dioxide substrate. By patterning the nickel and controlling the thickness of the layer using standard photolithographic techniques, they were able to grow an array of highly ordered, vertically aligned MWCNTs. In effect, this group exploited the high aspect ratio of CNTs to produce a nanoelectrode array. Capture oligonucleotides are then covalently bonded to the ends of the CNTs using carbodiimide chemistry and the hybridisation of the analyte sequences detected via measurement of mediated guanine oxidation. Using this approach, this group has demonstrated the detection of a DNA sequence related to the wild-type of the BRCA1 gene at sub-attomolar concentrations.

Aptamers present an attractive alternative to antibodies for biosensing applications as they demonstrate highly specific and selective binding, high stability and can be synthesised in a cost-effective manner [172,173]. As such, CNT-based aptasensors are an active field of research. Guo et al. [99] developed a method for the detection of the cardiac specific biomarker thrombin based on gold electrodes functionalised with a layer of 16-mercaptohexadecanoic acid (MHA). Aptamers are wrapped around SWCNTs in solution, bonded non-covalently via π–π interactions. The presence of the aptamer prevents the CNTs adsorbing to the MHA layer as a result of electrostatic repulsion. In the presence of the analyte, the aptamer binds preferentially with the analyte and the CNTs are free to adsorb to the MHA at the electrode surface, where they promote electron transfer between the electrode and the solution, leading to an increase in current. Using this method this group successfully detected thrombin to a limit of 50 pM. Su et al. [174] also developed a CNT-aptasensor from thrombin. By functionalising glassy carbon electrodes with a composite of MWCNTs and polyaniline and covalently binding thiolated aptamers to this composite, this group demonstrated the amperometric detection of thrombin to a limit of 80 fM. The binding of the protein to the aptamers depresses the electrochemical signal from the polyaniline.

Rostamabadi, et al. [175] developed a CNT-aptasensor by drop-casting a composite of SWCNTs and graphene oxide onto a glassy carbon electrode and forming gold nanoparticles on this composite via electroformation. Thiolated aptamers were bonded to the nanoparticles and the binding of the proteins to the aptamer detected via changes to the electrochemical impedance. This approached allowed for the detection of the breast cancer biomarker HER2 in serum samples with a detection limit of 50 fg/mL.

In addition to DNA, RNA, and protein detection, CNT-based DNA sensors have also been applied to the detection of metal ions. Zhang et al. [100] bonded cytosine rich oligonucleotides to SWCNTs via non-covalent attractions. These SWCNT/oligonucleotide complexes are then immobilised on an alkanethiol-functionalised gold electrode. The ionic charge of the oligonucleotide inhibits the electron transfer between the electrode and ferricyanide. In the presence of silver ions, the cytosine bases of the oligonucleotide form strong bonds with the ions, displacing the oligonucleotides from the SWCNTs and leading to an increase in the current from the reduction of ferricyanide. This approach enables the detection of silver ions to a detection limit of 1.5 nM. The electrochemical DNA sensors discussed in this section are summarised in Table 3 and the electrochemical aptasensors are summarised in Table 4.

#### 5.1.4. Non-Biomolecule-Based Electrochemical Sensors

Whilst the biomolecule-based sensors described in the preceding sections represent perhaps the most common examples of electrochemical CNT-based sensors, there are many examples of electrochemical CNT-based sensors that do not include biomolecules as recognition elements.

In recent years, there has been increasing interest in sensors based on molecularly imprinted polymers (MIPs). MIPs are synthetic analogues of antibodies. Very simply, polymers are synthesised containing the target molecule, bound either covalently or non-covalently to the polymer structure. The target molecule is then removed from the polymer matrix, leaving behind cavities bearing a particular shape that will allow the specific rebinding of the target molecule. In the presence of the target molecule in solution, the MIP will selectively bind to the target, in a manner analogous to antibody/antigen binding. MIPs are attractive candidates for the recognition elements of sensors as they are potentially more stable and cheaper to mass-produce than antibodies [176,177].

CNTs are of great interest for MIP applications as they can impart or enhance electrical conductivity to polymer composites, as well as improve their porosity and mechanical properties [178,179]. There are several examples of groups who have developed MIPs incorporating functionalised MWCNTs for the detection of analytes including epinephrine [178], the cardiac biomarker troponin T [179] and myoglobin [180]. These CNT-MIP sensors have been demonstrated to achieve detection at sub-nanogram [178] or sub-picogram [179] per millilitre concentrations.

CNTs have also been widely used in conjunction with other nanomaterials to functionalise electrode surfaces in order to improve the sensitivity of direct electrochemical detection for a variety of analytes. Noble metals such as gold and silver nanoparticles are a common example, having been used alongside CNTs to functionalise electrodes for the detection of analytes such as glucose [181], urea [182], bisphenol A [183] and volatile organic chemicals (VoCs) [184]. Nanoparticles based on graphene and graphene oxide are also commonly used alongside CNTs to improve electrode performance. Zhao et al. [185] used graphene oxide nanoparticles to prevent the aggregation of CNTs while developing electrodes for the detection of ascorbic acid, whereas Asadian et al. [186] employed CNTs as spacers when creating electrodes based on 3-dimensional graphene oxide networks for the detection of the anti-cancer drug Methotrexate. Huang et al. [187] developed an electrode comprised of a composite of graphene foam, CNTs and gold nanoparticles for the detection of dopamine and uric acid. Other examples of nanoparticles used in conjunction with CNTs for electrode functionalisation include nickel hydroxide nanosheets incorporated into a highly aligned CNT scaffold [188] and CNTs deposited onto copper nanowires [189].

Tan et al. [190] developed a dopamine sensor based on a hybrid MWCNT/boron-doped ultrananocrystalline diamond electrode. These microelectrodes have a 250 μm diameter, are fabricated via electrophoretic deposition and demonstrate sensitive dopamine detection in the presence of several common interfering compounds.

**Figure 5:**
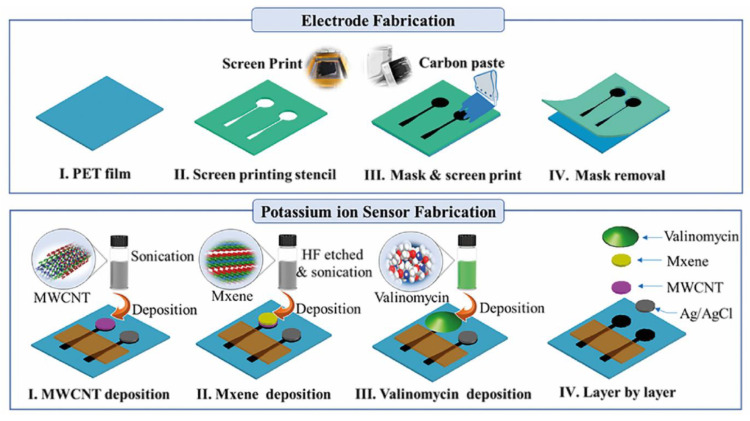
Fabrication process of MWCNT-MXene potassium ion sensors. Reprinted with permission from Zhang, S. et al., 2021 [191]. Copyright 2020 Elsevier B. V.

Zhang et al. [191] developed a sensor for analytes in human sweat based on MWCNTs and MXene-Ti_3_C_2_T_x_. Carbon paste electrodes were fabricated on a flexible polyethylene terephthalate (PET) substrate and MWCNTs drop-cast on the electrodes, followed by the MXene. An ion-selective valinomycin membrane was deposited over the electrode so as to selectively transport potassium ions to the electrode surface (Figure 5). In this manner, this group produced a flexible sensor that has been demonstrated for the selective measurement of potassium in human sweat using potentiometric measurement. This sensor has been integrated into a wearable, wireless and battery free device. The non-biomolecule based electrochemical sensors discussed in this section are summarised in Table 5.

### 5.2. Optical CNT Sensors

Optical transduction mechanisms are commonly used for biosensors as they are capable of high sensitivity, stability and are suitable for multiplex detection [192,193]. CNTs have fluorescent properties that are highly dependent on their physical structure, and consequently have been widely utilised in fluorescent sensors and biosensors [194].

A variety of biomolecules can be used to exploit the fluorescent properties of CNTs for biosensing applications. Strano et al. immobilised the enzyme luciferase to the surface of CNTs functionalised with phospholipids containing carboxylated poly (ethylene glycol). In the presence of adenosine triphosphate (ATP), the enzyme catalysed conversion of D-luciferin to oxyluciferin results in the quenching of the fluorescence of the CNTs, leading to a measurable change in the fluorescence intensity [195].

The same group reported the functionalisation of SWCNTs with bombolitin peptides in order to develop fluorescent sensors for nitroaromatic compounds. The changes to the secondary structure of the peptides caused by the binding of nitroaromatic compounds resulted in shifts in the fluorescent emission wavelength of the CNTs [196]. This group employed a similar approach by functionalising SWCNTs with oligonucleotides composed of repeating adenine (A) and thymine (T) bases via non-covalent attachment. This complex displays selectivity towards nitric oxide (NO) and the binding of NO results in the quenching of the CNTs’ fluorescence, allowing for the detection NO to a detection limit of 300 nM [197]. By dispersing SWCNTs in a chitosan film, in which antibodies are covalently bound to the chitosan network, Strano et al. developed a biosensor in which the binding of the protein troponin T—a cardiac specific biomarker—to the antibodies generated a shift in the intensity of the bandgap fluorescence of the SWCNTs. Using this approach, they reported a limit of detection of 100 ng/mL [198].

**Figure 6:**
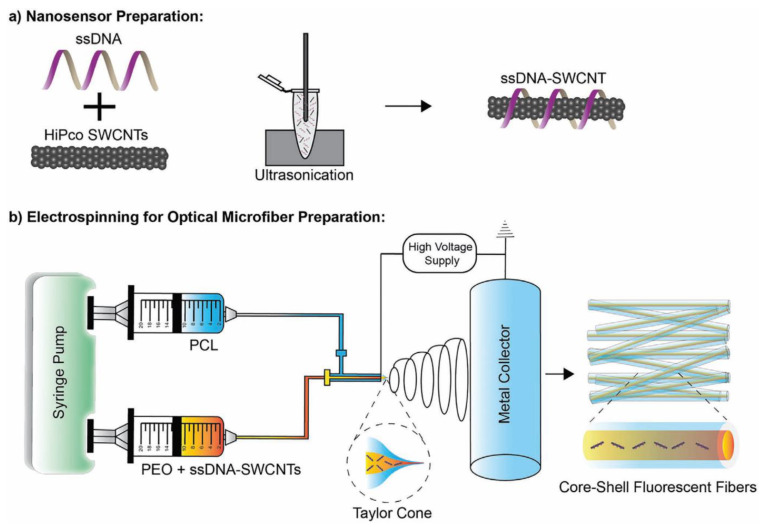
Fabrication of optical core-shell microfibers. (**a**) SWCNTs are dispersed by sonication in the presence of single stranded DNA (ssDNA). (**b**) The ssDNA-SWCNT dispersion is combined with poly (ethylene oxide) (PEO) and polycaprolactone (PCL) via electrospinning to create microfibers. Reprinted with permission from Safee, M.M. et al., 2021 [199]. Copyright 2021 Wiley-VCH GmbH.

Safaee et al. [199] incorporated CNTs into optical core-shell microfibrous textiles in order to create flexible fluorescent sensors. Single-stranded DNA (ssDNA) is non-covalently bound to SWCNTs and dispersed in an aqueous solution. The binding of the ionic DNA makes the combined ssDNA-SWCNT hydrophilic and thus more easily solubilised than native SWCNT. This dispersion is then combined with poly(ethylene oxide) (PEO) and polycaprolactone (PCL) via electrospinning to create core-shell microfibers (Figure 6). Changes to the fluorescent behaviour of these ssDNA-SWCNT nanosensors allows the detection of reactive oxygen species (specifically hydrogen peroxide). These core-shell microfibers have been incorporated into wound dressings for the purposes of real-time monitoring of wound healing.

CNTs can also be exploited as fluorescent quenchers. It is possible to label bio-recognition elements, such as oligonucleotides, with fluorophores. The oligonucleotides can then be bound non-covalently to CNTs such that the fluorophores are quenched by the CNTs via Förster resonance energy transfer (FRET). In the presence of the analyte molecules, the oligonucleotides will bind preferentially with the analyte, displacing the CNTs, leading to a significant increase in the fluorescent signal. Ma et al. [200] exploited this technique to achieve the detection of micro-RNA 155 (miRNA-155), a potential biomarker for breast cancer [201,202], with a detection limit of 33.4 fM and demonstrated successful detection in samples of human serum. Elmizadeh et al. [203] employed a similar technique using an aptamer of digoxin, a drug for congestive heart failure and arrhythmia, as the recognition element, achieving picomolar detection. Wang et al. [204] exploited this technique using a peptide probe as the biomolecule, and in this manner developed a fluorescent sensor for the detection of Cyclin A2, a prognostic indicator in early-stage cancer.

Surface plasmon resonance (SPR) is a highly sensitive optical technique widely employed for chemical and biological sensing applications. It exploits the formation of surface plasmon waves at the interface between metal surfaces, such as gold, and a dielectric by optical stimulation. Molecular binding at the metal surface is detected via changes to the refractive index of the system [192,193,205]. Lisi et al. [206] demonstrated the use of CNTs as signal amplifiers in SPR by binding them to label antibodies in a sandwich assay. Thus, they demonstrated the CNT amplified SPR detection of the Tau protein, an established biomarker for the diagnosis of Alzheimer’s disease, with a detection limit of 125 pM.

Lateral flow sensors have also been demonstrated using CNTs for visual detection. By binding antibody or oligonucleotide capture probes to the test strip and using CNT labelled capture probes in a sandwich assay, the accumulation of CNTs at the test site will result in a black band that can be observed visually. Qiu et al. [207] developed a DNA sensor using this approach. By binding biotin-functionalised capture oligonucleotides to the test strip via biotin/streptavidin linkages and covalently binding carboxyl functionalised CNTs to oligonucleotide label probes via carbodiimide bonds, they developed a lateral flow device oligonucleotide sensor with a detection limit of 0.1 nM using the naked eye. This limit can be improved to 40 pM using image processing software. Yao et al. [208] employed a similar method for heavy metal detection. By using thymine-rich capture and label oligonucleotides, they developed a lateral flow device for the detection of mercury ions in water with a naked-eye detection limit of 0.05 ppb.

Nandeshwar et al. [209] developed a novel colourimetric technique based upon the degradation of SWCNTs. The enzyme myeloperoxidase is an early warning biomarker for cardiovascular disease. In the presence of hydrogen peroxide and chloride ions, the enzyme will cause the oxidative degradation of SWCNTs. By measuring the change in optical contrast at near infrared wavelengths, this group has been able to detect myeloperoxidase with a detection limit of 327 ng/mL.

The fluorescent properties of CNTs have also been applied to the detection of COVID-19. By non-covalently binding the protein ACE2, which demonstrates a high binding affinity to the SARS-CoV-2 spike protein, Pinals et al. [210] demonstrated a simple and rapid fluorescent sensor for the SARS-CoV-2 spike protein. The binding of this protein to the ACE2 results in a 2-fold increase in the near infrared fluorescence. Using this method this group has successfully detected the spike protein and virus-like particles at a concentration of 35 mg/L, 5 s after exposure to the nanosensors. The optical biosensors discussed in this section are summarised in Table 6.

Their fluorescent properties make CNTs excellent labels for Raman spectroscopy as they have large Raman scattering cross-sections, simple spectra, and are more stable than many alternative Raman labels [211]. As such, CNTs have been employed as label molecules for Raman spectroscopy and surface enhanced Raman spectroscopy (SERS) [212,213,214,215]. However, as these are considered spectroscopy rather than biosensing applications, they will not be discussed any further in this review.

### 5.3. Field-Effect CNT Sensors

Field-effect transistors (FETs) are widely used microelectronic devices, generally consisting of three electrodes—the source, drain and gate electrodes—and a semi-conducting channel. The electrical current between the source and drain electrodes is dependent upon the voltage applied to the gate electrode. For sensing applications, the gate electrode is generally replaced by a combination of a reference electrode and a liquid medium. Changes to the electric field caused by the binding of charged molecules or changes to the ionic makeup at the surface of the channel will affect the charge carriers in the channel, thereby influencing the source/drain current. Variations of FETs have been widely applied in the field of biosensing utilising a variety of biorecognition elements. A variation of FETs is chemiresistive field-effect devices, which are essentially FETs without the gate electrode. The high conductivity and high surface area of CNTs make them well suited to field-effect devices, and as such field-effect based CNT biosensors has been an active area of research for many years [9,164,216,217,218].

**Figure 7:**
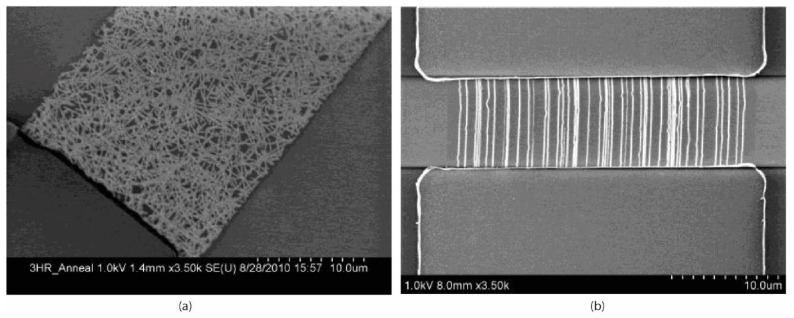
Scanning electron microscope images of: (**a**) SWCNT-based field-effect device in which the CNT element consists of randomly orientated networks of CNTs between the source and drain electrodes, and (**b**) SWCNT-based field-effect devices in which the CNTs are aligned such that they connect the source and drain electrodes [225].

The CNTs in field-effect biosensors are predominantly single-walled as SWCNTs are p-type semiconductors, and therefore ideally suited to form the necessary semi-conducting channel [216]. These CNTs are distributed between the source and drain electrodes of field-effect devices in one of two configurations: aligned between the electrodes [173,219,220,221] (Figure 7b), or in randomly orientated networks [101,222,223,224] (Figure 7a). Carbon nanotube network devices are fabricated by dispersing the CNTs in a solvent, such as dimethylformamide (DMF), and drop-casting the solution onto the substrate. This can either be performed between pre-fabricated electrodes or directly onto a substrate, with the electrodes fabricated on top using standard photolithographic techniques. Devices with aligned CNTs are commonly fabricated by drop-casting CNT dispersions between electrodes and performing dielectrophoresis (DEP) [219,220]. Alternatively, they can be fabricated by synthesising CNTs directly onto a substrate via chemical vapour deposition (CVD) [102,173].

CNT field-effect devices have been widely applied to the field of immunosensors. Kim et al. [222] developed a transistor-type field-effect immunosensor based on SWCNT networks. Antibodies are bound to the CNTs via a 1-pyrenebutanoic acid succinimidyl ester (PBASE) linker that binds non-covalently to CNTs through π-π interactions and binds covalently to the antibodies by amine groups. The binding of the analyte molecules to the antibodies changes the charge distribution at the FET surface, which results in a change in the source/drain conductivity. In this manner, Kim et al. have demonstrated detection of the PSA–ACT complex, a prostate cancer biomarker, to a detection limit of 1 ng/mL.

Oh et al. [223] demonstrated an FET biosensor for the amyloid-β protein—a biomarker for the early diagnosis of Alzheimer’s disease—featuring a gold gate electrode fabricated on top of a network of SWCNTs. Carbon nanotube channels are patterned using e-beam lithography and the gold electrodes—including source, drain and top gate electrodes—are fabricated on top of the CNTs using conventional photolithography techniques. The outer membranes of *E. coli* were immobilised on top of the gate electrode and antibodies bound to this membrane. The authors reported a detection limit for amyloid-β of 1 pg/mL.

The Mulchandani group reported many examples of chemiresistive field-effect biosensors based on aligned SWCNTs. By binding antibodies to the CNTs via the PBASE linker, they have developed immunosensors for the detection of *E. coli* 0157 and bacteriophage T7 [220]. By functionalising the SWCNTs with mercaptopropionic acid capped AuNPs and binding antibodies to the nanoparticles via a carbodiimide coupling reaction, they developed an immunosensor for the detection of the cardiac specific biomarker troponin-I [226]. By electrodepositing the conductive polymer poly (pyrrole-co-pyrrolepropylic acid) onto the SWCNTs and covalently binding antibodies to the polymer via pedant carboxyl groups, they have developed an immunosensor for the detection of the cardiac specific biomarker myoglobin in the ng/mL range [227].

The same group also employed an approach in which, rather than immobilise the antibody upon the sensor surface, the target molecule or an analogue thereof is immobilised upon the CNTs and the antibodies bound to these molecules. In the presence of the analyte, the antibodies will preferentially bind with the free-analyte, leading to the displacement of the antibodies from the sensor surface and resultant change in the charge distribution at the surface of the field-effect device [219,228]. This is an attractive approach when the target molecule is relatively small and/or weakly charged. The displacement of the antibody from the sensor surface will result in a greater change in the charge distribution than would the binding of the target molecule, thereby offering potentially greater sensitivities than a more conventional approach. Using this method, this group have reported the development of SWCNT-based field-effect sensors for the stress biomarker cortisol, with a detection limit of 1 pg/mL [219], and microcystin-LR (a toxin from bacteria in surface water) with a detection limit of 0.6 ng/mL [228].

Hwang et al. [229] recently reported the development of a chemiresistor device for the determination of tetrahydrocannabinol (THC) in breath. The device was reported to be able to be incorporated into a hand-held breathalyser. Enhanced selectivity toward THC over the more volatile breath components such as CO_2_, water, ethanol, methanol, and acetone were achievable by delaying the sensor reading to allow for the desorption of these compounds from the chemiresistor surface. Machine learning algorithms were utilised to improve the selective detection of THC with better accuracy at increasing quantities of THC. Commercial unsorted single-walled carbon nanotubes with 33% metallic and 67% semiconducting composition and poly (9,9-di-n-dodecylfluorenyl-2,7-diyl) (PFDD) wrapped enriched semiconducting single-walled carbon nanotubes with 0.1% metallic and 99.9% semiconducting composition were used in this study. Silicon dies with four pairs of interdigitated gold electrodes (IDE) were fabricated by photolithography and the SWCNTs were deposited as thin films on the interdigitated gold electrodes by dielectrophoresis. SWCNT suspensions were first dropped coated on top of the silicon dies and electrochemically deposited. The devices were rinsed with solvent and thermally annealed reportedly forming a thin SWCNT layer.

Other proteins have also been employed as biorecognition elements in CNT-field effect sensors. For example, Wasik et al. [230] have demonstrated a chemiresistive SWCNT biosensor for dengue virus using heparin. SWCNTs were drop-cast between microfabricated gold electrodes on substrate functionalised with 3-aminopropyltriethoxy silane to aid CNT immobilisation. The CNT networks were then non-covalently modified with 1-pyrenemethylamine to which heparin was subsequently covalently bonded via a carbodiimide linkage. The heparin acts as an analogue of the receptors of the virus during cell infection, thus resulting in the selective binding of the virus at the surface of the CNTs and a corresponding change in the surface charge distribution. Using this approach, the authors report a detection limit of approximately eight copies of the dengue virus per device.

With its polyionic backbone and highly specific hybridisation behaviour, DNA is ideally suited to act as the recognition element in field-effect sensors. Star et al. [101] have developed a field-effect transistor-based oligonucleotide biosensor using SWCNT networks. Capture oligonucleotides are bound to the CNTs via non-covalent aromatic interactions. The hybridisation of the analyte oligonucleotides results in a significant increase in charge at the sensor surface. Using this approach, the authors report a detection limit of 14 pM.

Ramani et al. [224] have reported a chemiresistive-field effect microRNA biosensor based upon the p19 protein immobilised on SWCNT networks. The p19 protein is covalently bound to CNTs deposited between two electrodes. The protein binds selectively to short (21–23 base pairs) double-stranded DNA, in the presence of the analyte sequence, complementary DNA (cDNA) probes in solution hybridise with analyte and the resultant duplex binds to the protein at the sensor surface, thereby increasing the charge. Using this method, the authors have successfully developed a sensor for miRNA122a—a potential biomarker for liver damage [231]—with a detection limit of 1 aM.

Fu et al. [102] demonstrated a chemiresistive field-effect biosensor based on aligned SWCNTs in which the detection mechanism functions by the displacement of oligonucleotide sequences. SWCNTs aligned between interdigitated electrodes are fabricated by a combination of contact printing and photolithography. Probe oligonucleotides drop cast onto the CNTs so that they are non-covalently attached via π-π interactions. In the presence of analyte oligonucleotides, the probe sequences will bind preferentially with the analyte, causing them to leave the surface of the field-effect device and resulting in a reduction in charge. By this approach, the authors have developed a biosensor capable of detecting viral DNA from the H5N1 virus (avian influenza) in the picomolar range.

Aptamers present an attractive alternative to antibodies for field-effect sensors as they are smaller in size, typically 1–2 nm compared to ca. 10 nm for antibodies. Significantly, they are generally smaller than the Debye length and therefore any changes in the charge distribution as a result of analyte binding will have a far greater influence upon the field-effect device [172,173].

Maehashi et al. [173] have developed a SWCNT-based field-effect transistor aptasensor for immunoglobin E, a biomarker of interest for immune deficiency-related disorders. The SWCNTs are formed and deposited via CVD and photolithography. The CNTs are functionalised with PBASE and amine-functionalised aptamers covalently bound via this linker. The changes in the charge distribution at the surface of the CNTs as the aptamer binds to analyte can be detected via the resultant changes to the source/drain current. In this manner immunoglobin E can be detected to a limit of 250 pM.

The group of Mulchandani have applied similar techniques from their efforts in immunosensors to aptasensors. By covalently binding aptamers to SWCNTs aligned by DEP and functionalised with PBASE, they have developed a chemiresistive aptasensor capable of detecting the protective antigen (PA) toxin of anthrax to a detection limit of 1 nM [232]. This group has also applied a displacement technique similar to that already mentioned for immunosensors. Oligonucleotides can be bound to CNTs via the PBASE linker, as already discussed, and aptamers partially hybridised to these oligonucleotides. In the presence of the analyte molecules, the aptamers will preferentially bind to the analyte, and therefore be displaced from the surface of the CNTs. As for immunosensors, this approach can produce far greater responses than the binding of small or weakly charged to immobilised aptamers [233].

This same group also applied oligonucleotide CNT-field effect devices to the detection of heavy metal ions. Thymine (T) nucleotides will form strong bonds to mercury ions [234]. By binding poly-T oligonucleotides to the CNTs of a field-effect device and hybridising these with poly-A oligonucleotides they have developed a chemiresistive biosensor for mercury ions. When mercury ions are present, they will bond with the poly-T sequences, displacing the poly-A sequences and resulting in a significant decrease in the charge at the CNT surface [235].

**Figure 8:**
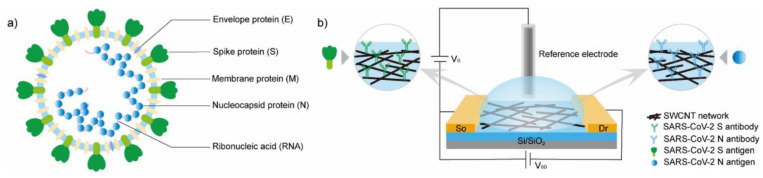
(**a**) Illustration of structure of SAR-CoV-2 and associated potential target proteins. (**b**) Illustration of SWCNT-based field-effect transistor (FET) sensor for detection of SARS-CoV-2 antigens. Reprinted with permission from Shao, W. et al., 2021 [236]. Copyright 2021 American Chemical Society.

Shao et al. [236] demonstrated a CNT-based FET sensor for the detection of the COVID-19 virus (SARS-CoV-2). SWCNTs were deposited between the source and drain electrodes using dielectrophoresis and antibodies covalently bound to the CNTs via NHS/EDC linkages (Figure 8). This group has investigated two relevant antibodies, the antibody for the spike protein (SAg) and the antibody for the nucleocapsid protein (NAg). This group has successfully demonstrated a proof of principle CNT-FET-based SARS-CoV-2, achieving detection limits of 0.55 fg/mL for SAg and 0.016 fg/mL for NAg in calibration samples.

Carbon nanotube-based FET biosensors are an area of particular interest as novel fabrication methodologies and materials offer the potential of devices that are low cost, flexible or mass-producible. Liang et al. [237] reported the fabrication of CNT-based FET biosensors on high-purity semiconducting CNT films with high uniformity on a wafer scale. This offers the potential of production on an industrial scale. Yi et al. [238] have demonstrated the fabrication of a whole CNT-based FET device using inkjet printing, using an ink containing SWCNTs and an M13 phage. The phage was genetically modified to produce a particular peptide sequence that demonstrates a strong binding affinity for SWCNTs [239]. Shen et al. [240] have developed a paper-based microfluidic FET-biosensor device. Antibodies are bound covalently to pyrene carboxylic acid functionalised SWCNTs, which are then deposited onto cellulose paper via inkjet printing to form FET devices. These devices have been demonstrated for the detection of human serum albumin (HSA) and human immunoglobin G (HIgG) with a detection limit of 1.5 pM. The field-effect biosensors discussed in this section are summarised in Table 7.

## 6. Challenges and Future Prospects

In the preceding sections, we have discussed how the physical and electronic properties of CNTs make them ideally suited to the development of biosensors. CNTs can also be combined with many other nanomaterials to form composite materials in order to enhance or exploit these characteristics. We have described how they can be functionalised with a variety of different biomolecules to develop sensors for the detection of a wide range of analytes using electrochemical, optical, and field-effect-based transduction mechanisms. CNT-based sensors have potential applications ranging from food safety to health monitoring, from counterterrorism to virus detection. However, despite three decades having passed since the first reporting of carbon nanotubes, few examples of CNT-based sensors have reached the market. There remain challenges to the widespread, real-world application of CNT-based sensors, primarily related to integration of CNT sensing elements into analytical devices and fabrication on an industrial scale.

In cases where CNTs are required to be synthesised in situ on an electrode surface or as part of a field-effect device, the synthesis procedure must be compatible with the materials of that device. For example, the arc discharge and laser ablation methods of CNT synthesis require high temperatures (1700 and 1200 °C, respectively [241]) which can damage many potential substrates. In comparison, PECVD synthesises CNTs at temperatures below 600 °C and is therefore more appropriate for the manufacture of CNT-based biosensing surfaces.

As discussed in Section 2, the chirality of CNTs can influence their electronic properties and this chirality can be difficult to control. This leads to issues of homogeneity and reproducibility for CNTs and CNT-based devices. Yield and purity are also of critical importance as small impurities or defects can dramatically alter the properties of CNTs, although these issues will affect SWCNTs more than MWCNTs. For the viable industrial-scale production of analytical devices, CNTs must be synthesised with reliable control of the physical and electrical properties in a highly reproducible manner. All of the main synthesis techniques require purification of the product [241] and further functionalisation.

As discussed previously, questions exist as to the toxicity and environmental impact of CNTs, with conflicting reports on the matter. If the commercial application of CNT-based biosensors, particularly in vivo devices, is to become widespread, these issues must be fully understood.

There have been many reports of CNTs being integrated with complementary metal-oxide-semiconductor (CMOS) electronics [242,243]. This presents a significant opportunity for the integration of CNT-based biosensors into existing hardware architectures. This is particularly true for FET-based sensors, which, as discussed, have the potential to be manufactured at large scale with high uniformity.

The coronavirus pandemic of the early 2020s will undoubtedly redouble research interest in rapid viral diagnostic techniques as well as areas such as point-of-care patient monitoring as the health services of the world adjust to the impact of the pandemic [244,245]. With their potential for developing biosensors with high-sensitivity and rapid response times, CNT-based analytical devices will no doubt play a major role in many of the responses to these challenges.

## Figures and Tables

**Table 1:** Summary of CNT electrochemical enzyme sensors. NA—not applicable, NR—not reported. * Unless otherwise stated.

Analyte	Enzyme	Method	Additional Nanomaterials	Limit of Detection (M) *	Detection Range (M) *	Reference
Glucose	Glucose oxidase (GOD)	Amperometry	NA	3 × 10^−4^	(1–15) × 10^−3^	[139]
Glucose	GOD	Amperometry	Platinum micropspheres	5 × 10^−5^	(0–5) × 10^−3^	[140]
Glucose	GOD	Amperometry	Graphene	2.99 × 10^−6^	(3–14) × 10^−3^	[143]
Glucose	GOD	Amperometry	NA	1 × 10^−5^	(0.05–13) × 10^−3^	[144]
Glucose	GOD	Amperometry	Platinum nanoparticles	5 × 10^−7^	(0.025–2000) × 10^−3^	[146]
Glucose	GOD	Amperometry	NA	8 × 10^−5^	(0–30) × 10^−3^	[150]
Alcohols	Alcohol dehydrogenase	Amperometry	NA	3.3 × 10^−3^	(12.5–100) × 10^−3^	[139]
Ethanol	Alcohol dehydrogenase	Amperometry	NA	1 × 10^−5^	(1–5) × 10^−4^	[148]
L-malic acid	Malate dehydrogenase	Amperometry	NA	6 × 10^−5^	(0–120) × 10^−6^	[141]
Xanthine	Xanthine oxidase	Amperometry	NA	1.2 × 10^−7^	(2–86) × 10^−6^	[142]
Choline	Choline oxidase	Amperometry	Gold nanoparticles	6 × 10^−7^	(3–120) × 10^−6^	[147]
Lead ions	Horseradish peroxidase (HRP)	Amperometry	NA	2.5 µg/L	0.092–0.55 mg/L	[149]
Copper ions	HRP	Amperometry	NA	4.2 µg/L	0.068–2 mg/L	[149]
Hydrogen peroxide	HRP	Amperometry	NA	5 × 10^−8^	(5–40) × 10^−6^	[151]
Hydrogen peroxide	HRP	Cyclic voltammetry	NA	NR	NR	[145]

**Table 2:** Summary of CNT electrochemical immunosensors. NA—not applicable, NR—not reported. * Unless otherwise stated.

Analyte	Method	Additional Nanomaterials	Limit of Detection (pg/mL) *	Detection Range (ng/mL)	Reference
Cardiac troponin T	Amperometry	NA	33	0.1–10	[155]
Dengue virus NS1 protein	Amperometry	NA	35,000	1000–2500	[156]
Zearalenone	Amperometry	Gold nanoparticles	0.15	0.001–0.1	[157]
Matrix metalloproteinase-3	Amperometry	NA	4	0.004–0.3	[158]
Interleukin 6	Amperometry	NA	0.5	NR	[159]
Prostate specific antigen	Amperometry	NA	5	NR	[163]
Interleukin 8	Amperometry	NA	8	NR	[163]
CA19-9	Square wave voltammetry	Magnetite nanoparticles	0.163	0.001–100	[162]
Human epidermal growth factor receptor 2	Impedance spectroscopy	Goldnanoparticles	7400	10–110	[160]
CA19-9	Impedance spectroscopy	NA	0.35 U/ml	NR	[161]

**Table 3:** Summary of CNT electrochemical DNA sensors. NA—not applicable, NR—not reported.

Analyte	Method	Additional Nanomaterials	Limit of Detection (fM)	Detection Range (M)	Reference
Anthrax lethal factor	Differential pulse voltammetry (DPV)	Copper oxide nanowires	3.5	10^−14^–10^−8^	[165]
Hepatitis B virus genomic DNA	DPV	Tungsten disulfide	2.5	10^−14^–10^−10^	[167]
Long non-coding RNAs	DPV	Gold nanocages	42.8	10^−14^–10^−7^	[168]
MicroRNA 21	DPV	Dendritic gold	0.01	10^−17^–10^−6^	[169]
Sequence specific to *E. coli*	DPV	NA	17 × 10^6^	NR	[98]
BRCA1 gene	Cyclic voltammetry	NA	NR	NR	[171]
Sequence specific to chronic myelogenous leukaemia	Impedance spectroscopy	NA	1	10^−15^–10^−6^	[166]

**Table 4:** Summary of CNT electrochemical aptasensors. NA—not applicable. * Unless otherwise stated.

Analyte	Method	Additional Nanomaterials	Limit of Detection (pM) *	Detection Range (nM) *	Reference
Thrombin	Differential pulse voltammetry	NA	0.08	0.001–4	[174]
Thrombin	Square wave voltammetry (SWV)	NA	50	0.5–10	[99]
Silver ions	SWV	NA	1500	2–100	[100]
Humna epidermal growth factor receptor 2	Impedance spectroscopy	Reduced graphene oxide; gold nanoparticles	50 fg/mL	0.1 pg/mL–1 ng/mL	[175]

**Table 5:** Summary of CNT non-biomolecule based electrochemical sensors. NA—not applicable, NR—not reported. * Unless otherwise stated.

Analyte	Method	Additional Nanomaterials	Limit of Detection (nM) *	Detection Range (µM) *	Reference
Glucose	Amperometry	Nickel hydroxide nanosheets	645	20–10,500	[188]
Glucose	Amperometry	Copper nanowires	0.33	10–2000	[189]
Glucose	Amperometry	Gold nanoparticles (AuNPs)	500	1–1000	[181]
Cardiac troponin T	Molecularly imprinted polymer (MIP)/Differential pulse voltammetry (DPV)	NA	0.04 pg/mL	0.1–8 pg/mL	[179]
Epinephrine	MIP/Differential pulse anodic stripping voltammetry	NA	0.02 ng/mL	0.09–5.9 ng/mL	[178]
Myoglobin	MIP/DPV	NA	9.7	0.06–6	[180]
Bisphenol A	DPV	AuNPs	4	0.01–0.7	[183]
Ascorbic acid	DPV	Graphene oxide/Goldnanorods	0.85	0.001–8000	[185]
Dopamine	DPV	3D graphene foam/AuNPs	1.36	0.1–48	[187]
Uric acid	DPV	3D graphene foam/AuNPs	33.03	0.5–60	[187]
Methotrexate	Cyclic voltammetry (CV)/DPV	Graphene	70	0.7–100	[186]
Urea	CV	Silver nanoparticles	4.7	0.066–20,600	[182]
3-ocatnone	CV	Gold-silver alloy nanoparticles	0.3 ppb	0–0.0025% (*v*/*v*)	[184]
Butanone	CV	Gold-silver alloy nanoparticles	0.5 ppb	0–0.055% (*v*/*v*)	[184]
Dopamine	CV/DPV	Boron-doped ultrananocrystalline diamond	9.5	0.033–1	[190]
Potassium ions	Potentiometry	MXene-Ti_3_C_2_T_x_	NR	1000–32,000	[191]

**Table 6:** Summary of CNT optical sensors. NA—not applicable, NR—not reported. * Unless otherwise stated.

Analyte	Method	Recognition Element	Limit of Detection (M) *	Detection Range (M) *	Reference
Adenosine triphosphate	Fluorescence	Luciferase	2.4 × 10^−7^	NR	[195]
Nitroaromatic compounds	Fluorescence	Bombolitin peptides	NR	NR	[196]
Nitric oxide	Fluorescence	Oligonucleotide sequence	3 × 10^−7^	NR	[197]
Troponin T	Fluorescence	Antibody	2.5 × 10^−9^	NR	[198]
Hydrogen peroxide	Fluorescence	NA	NR	5 × 10^−6^–5 × 10^−3^	[199]
MicroRNA 155	Fluorescence	DNA probe	3.34 × 10^−14^	1 × 10^−13^–1 × 10^−9^	[200]
Digoxin	Fluorescence	Aptamer	7.95 × 10^−12^	2.65 × 10^−11^–6.8 × 10^−10^	203]
Cyclin A2	Fluorescence	Peptide probe	5 × 10^−9^	NR	[204]
SARS-CoV-2 spike protein	Fluorescence	ACE2 protein	35 mg/L	NR	[210]
Tau protein	Surface plasmon resonance	Antibody	2 × 19^−9^	NR	[206]
DNA oligonucleotide	Lateral flow	DNA probe	1 × 10^−10^ (no instrumentation); 4 × 10^−11^ (instrumentation)	1 × 10^−10^–2 × 10^−8^	[207]
Mercury ions	Lateral flow	Oligonucleotide sequence	0.05 ppb	0.05–1 ppb	[208]
Myeloperoxidase	Optical contrast	NA	327 ng/mL	NR	[209]

**Table 7:** Summary of CNT field-effect sensors. NA—not applicable, NR—not reported.

Analyte	Method	Recognition Element	Limit of Detection	Detection Range	Reference
*E. coli* O157:H7	Chemiresistive	Antibody	10^5^ colony forming units (CFU)/mL (whole cell); 10^3^ CFU/mL (lysates)	10^3^–10^7^ CFU/mL	[220]
Bacteriophage T7	Chemiresistive	Antibody	10^3^ plaque forming units (PFU)/mL	10^2^–10^7^ PFU/mL	[220]
Cardiac troponin I	Chemiresistive	Antibody	0.01 ng/mL	0.01–10 ng/mL	[226]
Cardiac myoglobin	Chemiresistive	Antibody	1 ng/mL	1–1000 ng/mL	[227]
Cortisol	Chemiresistive	Antibody	1 pg/mL	0.001–10 ng/mL	[219]
Tetrahydrocannabinol	Chemiresistive	NA	0.163 ng	NR	[229]
Dengue virus (whole)	Chemiresistive	Heparin	8.4 × 10^2^ median tissue culture infectious dose (TCID_50_)/mL	8.4 × 10^2^–8.4 × 10^5^ TCID_50_/mL	[230]
Avian influenza virus (H5N1) DNA sequence	Chemiresistive	DNA probe	NR	2–200 pM	[102]
Anthrax protective antigen toxin	Chemiresistive	Aptamer	1 nM	1–400 nM	[232]
Adenosine triphosphate	Chemiresistive	Aptamer	1 pM	1–100 pM	[233]
Mercury ions	Chemiresistive	DNA probe	100 nM	100–1000 nM	[235]
PSA-ACT complex	Transistor	Antibody	1 ng/mL	1–100 ng/mL	[222]
Amyloid-beta	Transistor	Antibody	1 pg/mL	0.001–1 ng/mL	[223]
Microcystin-LR	Transistor	Antibody	0.6 pg/mL	0.001–1 ng/mL	[228]
Oligonucleotide (HFE gene)	Transistor	DNA probe	NR	NR	[101]
MicroRNA 122a	Transistor	p19 protein	1 aM	0.001–10 fM	[224]
Immunoglobulin E	Transistor	Aptamer	250 pM	0.25–20 nM	[173]
SARS-CoV-2 spike protein	Transistor	Antibody	0.55 fg/mL	0.0055–5.5 pg/mL	[236]
SARS-CoV-2 nucleocapsid protein	Transistor	Antibody	0.016 fg/mL	0.016–16 pg/mL	[236]

## References

[1] Saleh Ahammad A.J., Lee J.-J., Rahman M.A. (2009). Electrochemical sensors based on carbon nanotubes. Sensors.

[2] Yogeswaran U., Thiagarajan S., Chen S.-M. (2008). Recent updates of DNA incorporated in carbon nanotubes and nanoparticles for electrochemical sensors and biosensors. Sensors.

[3] Vashist S.K., Zheng D., Al-Rubeaan Luong J.H.T., Sheu F.-S. (2011). Advances in carbon nanotube based electrochemical sensors for bioanalytical applications. Biotechnol. Adv..

[4] Wang J. (2005). Carbon-nanotube based electrochemical biosensors: A review. Electroanalysis.

[5] Cha T.-G., Baker B.A., Sauffer M.D., Salgado J., Jaroch D., Rickus J.L., Porterfield D.M., Choi J.H. (2011). Optical nanosensors architecture for cell-signaling molecules using DNA aptamer-coated carbon nanotubes. ACS Nano.

[6] Chen Z., Zhang X., Yang R., Zhu Z., Chen Y., Tan W. (2011). Single-walled carbon nanotubes as optical materials for biosensing. Nanoscale.

[7] Bandaru N.M., Voelcher N.H. (2012). Glycoconjugate-functionalized carbon nanotubes in biomedicine. J. Mater. Chem..

[8] Wang X.-N., Hu P.-A. (2012). Carbon nanomaterials: Controlled growth and field-effect transistor biosensors. Front. Mater. Sci..

[9] Park R.S., Hills G., Sohn J., Mitra S., Shulaker M.M., Wong H.-S.P. (2017). Hysteresis-free carbon nanotube field-effect transistors. ACS Nano.

[10] Iijima S. (1991). Helical microtubules of graphitic carbon. Nature.

[11] Radushkevich L.V., Lukyanovich V.M. (1952). The structure of carbon forming in thermal decomposition of carbon monoxide on an iron catalyst. Russ. J. Phys. Chem..

[12] Oberlin A., Endo M., Koyama T. (1976). Filamentous growth of carbon through benzene decomposition. J. Cryst. Growth.

[13] Iijima S., Ichihashi T. (1993). Single-shell carbon nanotubes of 1-nm diameter. Nature.

[14] Zhao X., Ohkohchi M., Wang M., Iijima S., Ichihashi T., Ando Y. (1997). Preparation of high-grade carbon nanotubes by hydrogen arc discharge. Carbon.

[15] Rafique I., Kausar A., Anwar Z., Muhammad B. (2016). Exploration of epoxy resins, hardening systems, and epoxy/carbon nanotube composite designed for high performance materials: A review. Polym. Plast. Technol. Eng..

[16] Mahar B., Laslau C., Yip R., Sun Y. (2007). Development of carbon nanotube-based sensors—A review. IEEE Sens. J..

[17] Tilmaciu C.-M., Morris M.C. (2015). Carbon nanotube biosensors. Front. Chem..

[18] Nugent J.M., Santhanam K.S.V., Rubio A., Ajayan P.M. (2001). Fast electron transfer kinetics on multiwalled carbon nanotube microbundle electrodes. Nano Lett..

[19] Sayes C.M., Liang F., Hudson J.L., Mendez J., Guo W., Beach J.M., Moore V.C., Doyle C.D., West J.L., Billups W.E. (2006). Functionalization density dependence of single-walled carbon nanotubes cytotoxicity in vitro. Toxicol. Lett..

[20] Poland C.A., Duffin R., Kinloch I., Maynard A., Wallace W.A.H., Seaton A., Stone V., Brown S., MacNee W., Donaldson K. (2008). Carbon nanotubes introduced into the abdominal cavity of mice show asbestos-like pathogenicity in a pilot study. Nat. Nanotechnol..

[21] Dong L., Joseph K.L., Witkowski C.M., Craig M.M. (2008). Cytotoxicity of single-walled carbon nanotubes suspended in various surfactants. Nanotechnology.

[22] Laux P., Riebeling C., Booth A.M., Brain J.D., Brunner J., Cerrillo C., Creutzenberg O., Estrela-Lopis I., Geberl T., Johanson G. (2018). Challenges in characterizing the environmental fate and effects of carbon nanotubes and inorganic nanomaterials in aquatic systems. Environ. Sci. Nano..

[23] Prasek J., Drbohlavova J., Chomoucka J., Hubalek J., Jasek O., Adam V., Kizek R. (2011). Methods for carbon nanotubes synthesis—Review. J. Mater. Chem..

[24] Wang Y., Luc D., Wang F., Zhang D., Zhong J., Liang B., Guid X., Sun L. (2020). A new strategy to prepare carbon nanotube thin film by the combination of top-down and bottom-up approaches. Carbon.

[25] Li J., Ye Q., Cassell A., Tee Ng H., Stevens J., Han J., Meyyanppn M. (2003). Bottom-up approach for carbon nanotube interconnects. Appl. Phys. Lett..

[26] Guo T., Nikolaev P., Thess A., Colbert D.T., Smalley R.E. (1995). Catalytic growth of single-walled nanotubes by laser vaporization. Chem. Phys. Lett..

[27] Yudasaka M., Komatsu T., Ichihashi T., Achiba Y., Iijima S. (1998). Roles of laser light and heat in formation of single-wall carbon nanotubes by pulsed laser ablation of C_x_Ni_y_Co_y_ targets at high temperature. J. Phys. Chem. B.

[28] Zhang Y., Iijima S. (1998). Microscopic structure of as-grown single-wall carbon nanotubes by laser ablation. Philos. Mag. Lett..

[29] Zhang Y., Gu H., Iijima S. (1998). Single-wall carbon nanotubes synthesized by laser ablation in a nitrogen atmosphere. Appl. Phys. Lett..

[30] Li W.Z., Xie S.S., Qian L.X., Chang B.H., Zou B.S., Zhou R.A., Wang G. (1996). Large-scale synthesis of aligned carbon nanotubes. Science.

[31] Jose-Yacaman M., Terrones H., Rendon L., Dominguez J.M. (1995). Carbon structures grown from decomposition of a phenylacetylene and thiophene mixture on Ni nanoparticles. Carbon.

[32] Kumar M., Ando Y. (2010). Chemical vapor deposition of carbon nanotubes: A review on growth mechanism and mass production. J. Nanosci. Nanotechnol..

[33] Choi Y.C., Bae D.J., Lee Y.H., Lee B.S., Han I.T., Choi W.B., Lee N.S., Kim J.M. (2000). Low temperature synthesis of carbon nanotubes by microwave plasma-enhanced chemical vapor deposition. Synth. Met..

[34] Choi Y.C., Shin Y.M., Lee Y.H., Lee B.S. (2000). Controlling the diameter, growth rate, and density of vertically aligned carbon nanotubes synthesized by microwave plasma-enhanced chemical vapor deposition. Appl. Phys. Lett..

[35] Han J., Yang W.-S., Yoo J.-B. (2000). Growth and emission characteristics of vertically well-aligned carbon nanotubes grown on glass substrate by hot filament plasma-enhanced chemical vapor deposition. J. Appl. Phys..

[36] Murakami H., Hirakawa M., Tanaka C., Yamakawa H. (2000). Field emission from well-aligned, patterned, carbon nanotube emitters. Appl. Phys. Lett..

[37] Ren Z.F., Huang Z.P., Wang D.Z., Wen J.G. (1999). Growth of a single freestanding multiwall carbon nanotube on each nanonickel dot. Appl. Phys. Lett..

[38] Zhang Q., Yoon S.F., Ahn J., Gan B., Yu M.-B. (2000). Carbon films with high density nanotubes produced using microwave plasma assisted CVD. J. Phys. Chem. Solids.

[39] Tsai S.H., Chao C.W., Lee C.L., Shih H.C. (1999). Bias-enhanced nucleation and growth of the aligned carbon nanotubes with open ends under microwave plasma synthesis. Appl. Phys. Lett..

[40] Bell M.S., Teo K.B.K., Milne W.I. (2007). Factors determining properties of multi-walled carbon nanotubes/fibres deposited by PECVD. J. Appl. Phys. D.

[41] Amama P.B., Ogebule O., Maschmann M.R., Sands T.D., Fisher T.S. (2006). Dendrimer-assited low-temperature growth of carbon nanotubes by plasma-enhanced chemical vapor deposition. Chem. Commun..

[42] Gohier A., Djouadi M.A., Dubosc M., Granier A., Minea T.M., Sirhi L., Rossi F., Paredez P., Alvarez F. (2007). Single-and few-walled carbon nanotubes grown at temperatures as low as 450 °C: Electrical and field emission characterization. J. Nanosci. Nanotechnol..

[43] Kato T., Jeong G.H., Hirata T., Hatakeyama R., Tohji K., Motomiya K. (2003). Single-walled carbon nanotubes produced by plasma-enhanced chemical vapor deposition. Chem. Phys. Lett..

[44] Kyung S.J., Lee Y., Kim C.W., Lee J.H., Yeom G.Y. (2006). Field emission properties of carbon nanotubes synthesized by capillary type atmospheric pressure plasma enhanced chemical vapor deposition at low temperature. Carbon.

[45] Sato H., Hata K. (2006). Growth of carbon nanotubes by plasma-enhanced chemical vapor deposition. New Diam. Front. Carbon Technol..

[46] Shiratori Y., Hiraoka H., Yamamoto M. (2004). Vertically aligned carbon nanotubes produced by radio-frequency plasma-enhanced chemical vapor deposition at low temperature and their growth mechanism. Mater. Chem. Phys..

[47] Dubosc M., Minea T.M., Besland M.P., Cardinaud C., Granier A., Gohier A., Point S., Torres J. (2006). Low temperature plasma carbon nanotubes grown on patterned catalyst. Microelectron. Eng..

[48] Gohier A., Minea T.M., Djouadi M.A., Jimenez J., Granier A. (2007). Growth kinetics of low temperature single-wall and few walled carbon nanotubes grown by plasma enhanced chemical vapor deposition. Physica E.

[49] Ren J., Li F.-F., Lau J., Gonzalez-Urbina L., Licht S. (2015). One-pot synthesis of carbon nanofibers from CO2. Nano Lett..

[50] Pope C.J., Marr J.A., Howard J.B. (1993). Chemistry of fullerenes C60 and C70 formation in flames. J. Phys. Chem..

[51] Gore J.P., Sane A., Yellampalli S. (2011). Flame Synthesis of Carbon Nanotubes. Carbon Nanotubes—Synthesis, Characterization, Applications.

[52] Peigney A., Laurent C., Flahaut E., Bacsa R.R., Rousset A. (2001). Specific surface area of carbon nanotubes and bundles of carbon nanotubes. Carbon.

[53] Daniel S., Rao T.P., Rao K.S., Rani S.U., Naidu G.R.K., Lee H.Y., Kawai T. (2007). A review of DNA functionalized/grafted carbon nanotubes and their characterization. Sens. Actuators B.

[54] Zeng Y.L., Huang Y.F., Jiang J.H., Zhang X.B., Tang C.R., Shen G.L., Yu R.Q. (2007). Functionalization of multi-walled carbon nanotubes with poly(amidoamine) dendrimer for mediator free glucose biosensor. Electrochem. Commun..

[55] Chen R.J., Zhang Y., Wang D., Dai H. (2001). Noncovalent sidewall functionalization of single-walled carbon nanotubes for protein immobilization. J. Am. Chem. Soc..

[56] Yu J., Shapter J.G., Johnston M.R., Quinton J.S., Gooding J.J. (2007). Electron-transfer characteristics of ferrocene attached to single-walled carbon nanotubes (SWCNT) arrays directly anchored to silicon (100). Electrochim. Acta.

[57] Gong K., Yan Y., Zhang M., Su L., Xiong S., Mao L. (2005). Electrochemistry and electroanalytical applications of carbon nanotubes: A review. Anal. Sci..

[58] Moulton S.E., Minett A.I., Wallace G.G. (2005). Carbon nanotube based electronic and electrochemical sensors. Sens. Lett..

[59] Sanchez-Pomales G., Santiago-Rodriguez L., Cabrera C.R. (2009). DNA-funstionalized carbon nanotubes for biosensing applications. J. Nanosci. Nanotechnol..

[60] Santiago-Rodriguez L., Sanchez-Pomales G., Cabrera C.R. (2010). DNA-functionalized carbon nanotubes: Synthesis, self-assembly, and applications. Isr. J. Chem..

[61] Umasankar Y., Chen S.M. (2008). Recent trends in the application of carbon nanotubes-polymer composite modified electrodes for biosensors: A review. Anal. Lett..

[62] Fam D.W.H., Palaniappan A., Tok A.I.Y., Liedberg B., Moochhala S.M. (2011). A review on technological aspects influencing commercialization of carbon nanotube sensors. Sens. Act. B.

[63] Lei J., Ju H. (2010). Nanotubes in biosensing. Wiley Intrdiscip. Rev. Nanomed. Nanobiotechnol..

[64] Yeom S.H., Kang B.H., Kim K.J., Kang S.W. (2011). Nanostructures in biosensor—A review. Front. Biosci..

[65] Park K.C., Hayashi T., Tomiyasu H., Endo M., Dresselhaus M.S. (2005). Progressive and invasive functionalization of carbon nanotube sidewalls by diluted nitric acid under supercritical conditions. J. Mater. Chem..

[66] Park T.J., Banerjee S., Hemraj-Benny T., Wong S.S. (2006). Purification strategies and purity visualization techniques for single-walled carbon nanotubes. J. Mater. Chem..

[67] Bergeret C., Cousseau J., Fernandez V., Mevellec J.-Y., Lefrant S. (2008). Spectroscopic evidence of carbon nanotubes’ metallic character loss induced by covalent functionalization via nitric acid purification. J. Phys. Chem..

[68] Gromov A., Dittmer S., Svensson J., Nerushev O.A., Perez-Garcia S.A., Licea-Jimenez L., Rychwalski R., Campbell E.E.B. (2005). Covalent amino-functionalisation of single-wall carbon nanotubes. J. Mater. Chem..

[69] Karousis N., Tagmatarchis N., Tasis D. (2010). Current progress on the chemical modification of carbon nanotubes. Chem. Rev..

[70] Campidelli S. (2011). Click chemistry for carbon nanotubes functionalization. Curr. Org. Chem..

[71] Campidelli S., Ballesteros B., Filoramo A., Diaz-Diaz D., de la Torre G., Torres T., Rahman G.M.A., Ehli C., Kiessling D., Werner F. (2008). Facile decoration of functionalized single-wall carbon nanotubes with phthalocyanines via “click chemistry”. J. Am. Chem. Soc..

[72] Clave G., Campidelli S. (2011). Efficient covalent functionalization of carbon nanotubes: The use of “click chemistry”. Chem. Sci..

[73] Coates M., Griveau S., Bedioui F., Nyokong T. (2012). Layer by layer electrode surface functionalization using carbon nanotubes, electrochemical grafting of azide-alkyne functions and click chemistry. Electroanalysis.

[74] Guo Z., Liang L., Liang J.J., Ma Y.-F., Yang X.-Y., Ren D.-M., Chen Y.-S., Zheng J.-Y. (2008). Covalently beta-cyclodextrin modified single-walled carbon nanotubes: A novel artificial receptor synthesized by ‘click’ chemistry. J. Nanopart. Res..

[75] Han J., Gao C. (2010). Functionalization of carbon nanotubes and other nanocarbons by azide chemistry. Nano-Micro Lett..

[76] Jing L., Liang C., Shi X., Ye S., Xian Y. (2012). Fluorescent probe for Fe(III) based on pyrene grafted multiwalled carbon nanotubes by click reaction. Analyst.

[77] Kumar I., Rana S., Rode C.V., Cho J.W. (2008). Functionalization of single-walled carbon nanotubes with azides derived from amino acids using click chemistry. J. Nanosci. Nanotechnol..

[78] Li H., Cheng F., Duft A.M., Adronov A. (2005). Functionalization of single-walled carbon nanotubes with well-defined polystyrene by “Click” coupling. J. Am. Chem. Soc..

[79] Liu J., Nie Z., Cao Y., Adronov A.L.H., Li H. (2008). “Click” coupling between alkyne-decorated multi-walled carbon nanotubes and reactive PDMA-PNIPAM micelles. J. Polym. Sci. A.

[80] Palacin T., Le Khanh H., Jousselme B., Jegou P., Filoramo A., Ehli C., Guldi D.M., Campidelli S. (2009). Efficient functionalization of carbon nanotubes with porphyrin dendrons via click chemistry. J. Am. Chem. Soc..

[81] Qi H., Ling C., Huang R., Qiu X., Shangguan L., Gao Q., Zhang C. (2012). Functionalization of single-walled carbon nanotubes with protein by click chemistry as sensing platform for sensitized electrochemical immunoassay. Electrochim. Acta.

[82] Rana S., Kumar I., Yoo H.J., Cho J.W. (2009). Assembly of gold nanoparticles on single-walled carbon nanotubes by using click chemistry. J. Nanosci. Nanotechnol..

[83] Tuci G., Vinattieri C., Luconi L., Ceppatelli M., Cicchi S., Brandi A., Filippi J., Melucci M., Giambastiani G. (2012). “Click” on tubes: A versatile approach towards multimodal functionalization of SWCNTs. Chem. Eur. J..

[84] Hetemi D., Noel V., Pinson J. (2020). Grafting of diazonium salts on surfaces: Application to biosensors. Biosensors.

[85] Gautier C., Lopez I., Breton T. (2021). A post-functionalization toolbox for diazonium (electro)-grafted surfaces: Review of the coupling methods. Mater. Adv..

[86] Polsky R., Harper J.C., Wheeler D.R., Dirk S.M., Arango D.C., Brozik S.M. (2008). Electrically addressable diazonium-functionalized antibodies for multi-analyte electrochemical sensor applications. Biosens. Bioelectron..

[87] Kim J.H., Lee M.J., Park E.J., Lee J.-Y., Lee C.J., Min N.K. (2012). Plasma processing: Technology for the batch fabrication of carbon nanotube film electrodes for biointerfaces. Plasma Process. Polym..

[88] Chen C., Liang B., Lu D., Ogino A., Wang X., Nagatsu M. (2010). Amino group introduction onto multiwall carbon nanotubes by NH3/Ar plasma treatment. Carbon.

[89] Park E.J., Lee J.Y., Kim J.H., Kim S.K., Lee C.J., Min N.K. (2010). Electrochemical characterization of O2 plasma functionalized multi-walled carbon nanotube electrode for legionella pneumophila DNA sensor. Jpn. J. Appl. Phys..

[90] Lee J.Y., Park E.J., Lee C.J., Kim S.W., Pak J.J., Min N.K. (2009). Flexible electrochemical biosensors based on O2 plasma functionalized MWCNT. Thin Solid Films.

[91] Wang S.C., Chang K.S., Yuan C.J. (2009). Enhancement of electrochemical properties of screen-printed carbon electrodes by oxygen plasma treatment. Electrochim. Acta.

[92] Yeo S., Choi C., Jang C.W., Lee S., Jhon W.M. (2013). Sensitivity enhancement of carbon nanotube-based ammonium ion sensors through surface modification by using oxygen plasma treatment. Appl. Phys. Lett..

[93] Luais E., Thobie-Gautier C., Tailleur A., Djouadi M.-A., Granier A., Tessier P.Y., Debarnot D., Poncin-Epaillard F., Boujita M. (2010). Preparation and modification of carbon nanotubes by cold plasma processes toward the preparation of amperometric biosensors. Electrochim. Acta.

[94] Huber T.A., Kopac M.C., Chow C. (2008). The quantitative removal of metal catalyst from multi-walled carbon nanotubes with minimal tube damage. Can. J. Chem..

[95] Ying L.S., bin Mohd Salleh M.A., Yusoff H.M., Abul Rashid S.B.B., Razak J.A. (2011). Continuous production of carbon nanotubes —A review. J. Ind. Eng. Chem..

[96] Ding X., Li H., Deng L., Peng Z., Chen H., Wang D. (2011). A novel homogenous detection method based on the self-assembled DNAzyme labeled DNA probes with SWCNT conjugates and its application in detecting pathogen. Biosens. Bioelectron..

[97] Zhang Y., Li B., Yan C., Fu L. (2011). One-pot fluorescence detection of multiple analytes in homogenous solution based on noncovalent assembly of single-walled carbon nanotubes and aptamers. Biosens. Bioelectron..

[98] Ozkan-Ariksoysal D., Kayran Y.U., Yilmaz F.F., Ciucu A.A., David I.G., David V., Hosgor-Limoncu M., Ozsoz M. (2017). DNA-wrapped multi-walled carbon nanotube modified electrochemical biosensor for the detection of Escherichia coli from real samples. Talanta.

[99] Guo K., Wang Y., Chen H., Ji J., Zhang S., Kong J., Liu B. (2011). An aptamer-SWCNT biosensor for sensitive detection of protein via mediated signal transduction. Electrochem. Commun..

[100] Zhang Z., Yan J. (2014). A signal-on electrochemical biosensor for sensitive detection of sliver ion based on alkanethiol-carbon nanotube-oligonucleotide modified electrodes. Sens. Actuators B.

[101] Star A., Tu E., Niemann J., Gabriel J.-C.P., Joiner C.S., Valcke C. (2006). Label-free detection of DNA hybridization using carbon nanotube network field-effect transistors. Proc. Natl. Acad. Sci. USA.

[102] Fu Y., Romay V., Liu Y., Ibarlucea B., Baraban L., Khavrus V., Oswald S., Bachmatiuk A., Ibrahim I., Rummeli M. (2017). Chemiresitive biosensors based on carbon nanotubes for label-fee detection of DNA sequences derived from avian influenza virus H5N1. Sens. Acutators B.

[103] Abdula D., Nguyen K.T., Shim M. (2007). Raman, spectral evolution in individual metallic single-walled carbon nanotubes upon covalent sidewall functionalization. J. Phys. Chem. C.

[104] de Fuentes O.A., Ferri T., Frasconi M., Paolini V., Santucci R. (2011). Highly ordered covalent anchoring of carbon nanotubes on electrode surfaces by diazonium salt reactions. Angew. Chem. Ind. Ed..

[105] Balasubramanian K., Burghard M., Kern K. (2008). Effect of the electronic structure of carbon nanotubes on the selectivity of electrochemical functionalization. Phys. Chem. Chem. Phys..

[106] Chakraborty A.K., Coleman K.S., Dhanak V.R. (2009). The electronic fine structure of 4-nitrophenyl functionalized single-walled carbon nanotubes. Nanotechnology.

[107] Ellison M.D., Gasda P.J. (2008). Functionalization of single-walled carbon nanotubes with 1,4-benzenediamine using a diazonium reaction. J. Phys. Chem. C.

[108] Fantini C., Usrey M.L., Srano M.S. (2007). Investigation of electronic and vibrational properties of single-walled carbon nanotubes functionalized with diazonium salts. J. Phys. Chem. C.

[109] Le Floch F., Thuaire A., Bidan G., Simonato J.P. (2009). The electrochemical signature of functionalized single-walled carbon nanotubes bearing electroactive gaps. Nanotechnology.

[110] Gohier A., Nekelson F., Helezen M., Jegou P., Deniau G., Palacin S., Mayne-L’Hermite M. (2011). Tunable grafting of functional polymers onto carbon nanotubes using diazonium chemistry in aqueous media. J. Mater. Chem..

[111] Gross M.L., Hickner M.A. (2010). Using cyclic voltammetry to measure bandgap modulation of functionalized carbon nanotubes. Electrochem. Solid State Lett..

[112] Karousis N., Ali-Boucetta H., Kostarelos K., Tagmatarchis N. (2008). Aryl-derivatized, water-soluble functionalized carbon nanotubes for biomedical applications. Mater. Sci. Eng. B.

[113] Kim S.K., Jeon S. (2012). Improved electrocatalytic effect of carbon nanomaterials by covalently anchoring with CoTAPP via diazonium salt reactions. Electrochem. Commun..

[114] Lipinska M.E., Rebelo S.L.H., Pereira M.F.R., Gomes J.A.N.F., Freire C., Figueiredo J.L. (2012). New insights into the functionalization of multi-walled carbon nanotubes with aniline derivatives. Carbon.

[115] Mevellec V., Roussel S., Tessier L., Chancolon J., Mayne-L’Hermite M., Deniau G., Viel P., Palacin S. (2007). Grafting polymers on surfaces, a new powerful and versatile diazonium salt-based one-step process in aqueous media. Chem. Mater..

[116] Ungureanu E.M., Pilan L., Mehea A., Le Floch F., Simonato J.P., Bidan G. (2008). Preliminary tests for grafting p-nitrophenyl on carbon nanotubes. Rev. Chim..

[117] Bahr J.L., Yang J., Kosynkin D.V., Bronikowski M.J., Smalley R.E., Tour J.M. (2001). Functionalization of carbon nanotubes by electrochemical reduction of aryl diazonium salts: A bucky paper electrode. J. Am. Chem. Soc..

[118] Downard A.J. (2000). Electrochemically assisted covalent modification of carbon electrodes. Electroanalysis.

[119] Pinson J., Podvorica F. (2005). Attachment of organic layers to conductive or semiconductive surfaces by reduction of diazonium salts. Chem. Soc. Rev..

[120] Combellas C., Delamar M., Kanoufi F., Pinson J., Podvorica F.I. (2005). Spontaneous grafting of iron surfaces by reduction of aryldiazonium salts in acidic or neutral aqueous solution. Application to the protection of iron against corrosion. Chem. Mater..

[121] Doyle C.D., Rocha J.D.R., Weisman R.B., Tour J.M. (2008). Structure-dependent reactivity of semiconducting single-walled carbon nanotubes with benzenediazonium salts. J. Amer. Chem. Soc..

[122] Brooksby P.A., Downard A.J. (2005). Multilayer nitroazobenzene films covalently attached to carbon. An AFM and electrochemical study. J. Phys. Chem. B.

[123] Ko J.W., Woo J.M., Jinhong A., Cheon J.H., Lim J.H., Kim S.H., Chun H., Kim E., Park Y.J. (2011). Multi-order dynamic range DNA sensor using a gold decorated SWCNT random network. ACS Nano.

[124] Wei G., Pan C., Reichert J., Jandt K.D. (2010). Controlled assembly of protein-protected gold nanoparticles on noncovalent functionalized carbon nanotubes. Carbon.

[125] Zhou J., Barbara P., Paranjape M. (2010). Novel in-situ decoration of single-walled carbon nanotube transistors with metal nanoparticles. J. Nanosci. Nanotechnol..

[126] Lai Y., Bai J., Shi X., Zeng Y., Xian Y., Hou J., Jin L. (2013). Graphene oxide as nanocarrier for sensitive electrochemical immunoassay of clenbuterol based on labeling amplification strategy. Talanta.

[127] Pan Y., Zhang Y.Z., Li Y. (2013). Layer-by-layer self-assembled multilayer films of single-walled carbon nanotubes and tin disulfide nanotparticles with chitosan for the fabrication of biosensors. J. Appl. Polym. Sci..

[128] Siqueria J.R., Gabriel R.C., Zucolotto V., Silva A.C.A., Dantas N.O., Gasparotto L.H.S. (2012). Electrodeposition of catalytic and magnetic gold nanoparticles on dendrimer-carbon nanotube layer-by-layer films. Phys. Chem. Chem. Phys..

[129] Yu A., Wang Q., Yong J., Mahon P.J., Malherbe F., Wang F., Zhang H., Wang J. (2012). Silver nanoparticle-carbon nanotube hybrid films: Preparation and electrochemical sensing. Electrochim. Acta.

[130] Flavel B.S., Yu J., Ellis A.V., Quinton J.S., Shapter J.G. (2008). Solution chemistry approach to fabricate vertically aligned carbon nanotubes on gold wires: Towards vertically integrated electronics. Nanotechnology.

[131] Chu X., Duan D., Shen G., Yu R. (2007). Amperometric glucose biosensor based on electrodeposition of platinum nanoparticles onto covalently immobilized carbon nanotube electrode. Talanta.

[132] Lee K.P., Gopalan A.I., Santhosh P., Manesh K.M., Kim J.H., Kim K.S. (2006). Fabrication and electrocatalysis of self-assembly directed gold nanoparticles anchored carbon nanotubes modified electrode. J. Nanosci. Nanotechnol..

[133] Rao S.G., Huang L., Murray J. (2011). Assembly of single-walled carbon nanotubes on patterns of Au nanoparticles. Appl. Surf. Sci..

[134] Villalonga R., Diez P., Eguilaz M., Martinez P., Pingarron J.M. (2012). Supramolecular immobilization of xanthine oxidase on electropolymerised matrix of functionalized hybrid gold nanoparticles/single-walled carbon nanotubes for the preparation of electrochemical biosensors. ACS Appl. Mater. Interfaces.

[135] Clark L.C., Lyons C. (1962). Electrode systems for continuous monitoring in cardiovascular surgery. Ann. N. Y. Acad. Sci..

[136] Hammond J.L., Formisano N., Estrela P., Carrara S., Tkac J. (2016). Electrochemical biosensors and nanobiosensors. Essays Biochem..

[137] Alim S., Vejayan J., Yusoff M.M., Kafi A.K.M. (2018). Recent uses of carbon nanotubes & gold nanoparticles in electrochemistry with application in biosensing: A review. Biosens. Bioelectron..

[138] Sanati A., Jalali M., Raeissi K., Karimzadeh K., Kharaziha M., Mahshid S.S. (2019). A review on recent advancements in electrochjemical biosensing using carbonaceous nanomaterials. Microchim. Acta.

[139] Zappi D., Caminiti R., Ingo G.M., Sadun C., Tortolini C., Antonelli M.L. (2017). Biologically friendly room temperature ionic liquids and nanomaterials for the development of innovative enzymatic biosensors. Talanta.

[140] Chen C., Ran R., Yang Z., Lv R., Shen W., Kang F., Huang Z.-H. (2018). An efficient flexible electrochemical glucose sensor based on carbon nanotubes/carbonized silk fabrics decorated with Pt microspheres. Sens. Actuators B.

[141] Ruhal A., Rana J.S., Kumar S., Kumar A. (2012). Immobilization of malate dehydrogenase on carbon nanotubes for development of malate biosensor. Cell. Mol. Biol..

[142] Dervisevic M., Custiuc E., Cevik E., Senel M. (2015). Construction of novel xanthine biosensor using polymeric mediator/MWCNT nanocomposite layer for fish freshness detection. Food Chem..

[143] Madhurantakam S., Babu K.J., Rayappan J.B.B. (2017). Fabrication of mediator-free hybrid nano-interfaced electrochemical biosensor for monitoring cancer cell proliferation. Biosens. Bioelectron..

[144] Wang S.G., Zhang Q., Wang R., Yoon S.F., Ahn J., Yang D.J., Tian J.Z., Li J.Q., Zhou Q. (2003). Multi-walled carbon nanotubes for the immobilization of enzyme in glucose biosensors. Electrochem. Commun..

[145] Lee Y.-M., Kwon O.-Y., Yoon Y.-J., Ryu K. (2006). Immobilization of horseradish peroxidase on multi-wall carbon nanotubes and its electrochemical properties. Biotechnol. Lett..

[146] Hrapovic S., Liu Y., Male K.B., Luong J.H.T. (2004). Electrochemical biosensing platforms using platinum nanoparticles and carbon nanotubes. Anal. Chem..

[147] Magar H.S., Ghica M.E., Abbas M.N., Brett C.M.A. (2017). A novel sensitive amperometric choline biosensor based on multiwalled carbon nanotubes and gold nanoparticles. Talanta.

[148] Wilson T.A., Musameh M., Kyratzis I.L., Zhang J., Bond A.M., Hearn T.W. (2017). Enhanced NADH oxidation using polytyramine/carbon nanotube modified electrodes for ethanol biosensing. Electroanalysis.

[149] Moyo M., Okonkwo J.O., Agyei N.M. (2014). An amperometric biosensor based on horseradish peroxidase immobilized onto maize tassel-multi-walled carbon nanotubes modified glassy carbon electrode for determination of heavy metal ions in aqueous solution. Enzyme Microb. Technol..

[150] Lin Y., Lu F., Tu Y., Ren Z. (2004). Glucose biosensors based on carbon nanotube nanoelectrode ensembles. Nano Lett..

[151] Yu X., Chattopadhyay D., Galeska I., Papadimitrakopoulos F., Rusling J.F. (2003). Peroxidase activity of enzymes bound to the ends of single-wall carbon nanotube forest electrodes. Electrochem. Commun..

[152] Liu J., Chou A., Rahmat W., Paddon-Row M.N., Gooding J.J. (2005). Achieving direct electrical connection to glucose oxidase using aligned single walled carbon nanotube arrays. Electroanalysis.

[153] Tasca F., Harreither W., Ludwig R., Gooding J.J., Gorton L. (2011). Cellobiose dehydrogenase aryl diazonium modified single walled carbon nanotubes: Enhanced direct electron transfer through a positively charged surface. Anal. Chem..

[154] Felix F.S., Angnes L. (2018). Electrochemical immunosensors—a powerful tool for analytical applications. Biosens. Bioelectron..

[155] Gomes-Filho S.L.R., Dias A.C.M.S., Silva M.M.S., Silva B.V.M., Dutra R.F. (2013). A carbon nanotube-based electrochemical immunosensor for cardiac troponin T. Microchem. J..

[156] Silva M.M.S., Dias A.C.M.S., Silva B.V.M., Gomes-Filho S.L.R., Kubota L.T., Goulart M.O.F., Dutra R.F. (2015). Electrochemical detection of dengue virus NS1 protein with a poly(allylsamine)/carbon nanotube layered immunoelectrode. J. Chem. Technol. Biotechnol..

[157] Riberi W.I., Tarditto L.V., Zon M.A., Arevalo F.J., Fernandez H. (2018). Development of an electrochemical immunosensor to determine zearalenone in maize using carbon screen printed electrodes modified with multi-walled carbon nanotubes/polyethyleneimine dispersions. Sens. Actuators B.

[158] Munge B.S., Fisher J., Millord L.N., Krause C.E., Dowd R.S., Rusling J.F. (2010). Sensitive electrochemical immunosensor for matrix metalloproteinase-3 based on single-wall carbon nanotubes. Analyst.

[159] Malhotra R., Patel V., Vaque J.P., Gutkind J.S., Rusling J.F. (2010). Ultrasensitive electrochemical immunosensor for oral cancer biomarker IL-6 using carbon nanotube forest electrodes and multilabel amplification. Anal. Chem..

[160] Arkan E., Saber R., Karimi Z., Shamsipur M. (2015). A novel antibody-antigen based impedimetric immunosensor for low level detection of HER2 in serum samples of breast cancer patients via modification of a gold nanoparticles decorated multiwall carbon nanotube-ionic liquid electrode. Anal. Chim. Acta.

[161] Thapa A., Soares A.C., Soares J.C., Awan I.T., Volpati D., Melendez M.E., Fregnani J.H.T.G., Carvalho A.L., Oliveira O.N. (2017). Carbon nanotube matrix for highly sensitive biosensors to detect pancreatic cancer biomarker CA19-9. ACS Appl. Mater. Interfaces.

[162] Kalyani T., Sangili A., Nanda A., Prakash S., Kaushik A., Jana S.K. (2021). Bio-nanocomposite based highly sensitive and label-free electrochemical immunosensor for endometriosis diagnostics application. Bioelectrochemistry.

[163] Wan Y., Deng W., Su Y., Zhu X., Peng C., Hu H., Peng H., Song S., Fan C. (2011). Carbon nanotube-based ultrasensitive multiplexing electrochemical immunosensor for cancer biomarkers. Biosens. Bioelectron..

[164] Ferrier D.C., Shaver M.P., Hands P.J.W. (2015). Micro-and nanostructure-based oligonucleotide sensors. Biosens. Bioelectron..

[165] Chen M., Hou C., Huo D., Yang M., Fa H. (2016). An ultrasensitive electrochemical DNA biosensor based on a copper oxide nanowires/single-walled carbon nanotube nanocomposite. Appl. Surf. Sci..

[166] Ghrera A.S., Pandey C.M., Malhotra B.D. (2018). Multiwalled carbon nanotube modified microfluidic-based biosensor chip for nucleic acid detection. Sens. Actuators B.

[167] Liu X., Shuai H.-L., Liu Y.-J., Huang K.-J. (2016). An electrochemical biosensor for DNA detection based on tungsten disulfide/multi-walled carbon nanotube composites and hybridization chain reaction amplification. Sens. Actuators B.

[168] Chen M., Wu D., Tu S., Yang C., Chen D., Xu Y. (2021). A novel biosensor for the ultrasensitive detection of the lncRNA biomarker MALAT1 in non-small cell lung cancer. Sci. Rep..

[169] Sabahi A., Salahandish R., Ghaffarinejad A., Omidinia E. (2020). Electrochemical nano-genosensor for highly sensitive detection of miR-21 biomarker based on SWCNT-grafted dendritic Au nanostructure for early detection of prostate cancer. Talanta.

[170] Larrea E., Sole C., Manterola L., Goicoechea I., Armesto M., Arestin M., Caffarel M.M., Araujo A.M., Araiz M., Fernandez-Mercado M. (2016). New concepts in cancer biomarkers: Circulating miRNAs in liquid biopsies. Int. J. Mol. Sci..

[171] Li J., Ng H.T., Cassell A., Fan W., Chen H., Ye Q., Koehne J., Han J., Meyyappan M. (2003). Carbon nanotube nanoelectrode array for ultrasensitive DNA detection. Nano Lett..

[172] So H.-M., Won K., Kim Y.H., Kim B.-K., Ryu B.H., Na P.S., Kim H., Lee J.-O. (2005). Single-walled carbon nanotube biosensors using aptamers as molecular recognition elements. J. Am. Chem. Soc..

[173] Maehashi K., Katsura T., Kerman K., Takamura Y., Matsumoto K., Tamiya E. (2007). Label-free protein biosensor based on aptamer-modified carbon nanotube field-effect transistors. Anal. Chem..

[174] Su Z., Xu X., Xu H., Zhang Y., Li C., Ma Y., Song D., Xie Q. (2017). Amperometric thrombin aptasensor using a glassy carbon electrode modified with polyaniline and multiwalled carbon nanotubes tethered with a thiolated aptamer. Microchim. Acta.

[175] Rostamabadi P.F., Heydari-Bafrooei E. (2019). Impedimetrc aptasensing of the breast cancer biomarker HER2 using a glassy carbon electrode modified with gold nanoparticles in a composite consisting of electrochemically reduced graphene oxide and single-walled carbon nanotubes. Microchim. Acta.

[176] Beyazit S., Bui B.T.S., Haupt K., Gonzato C. (2016). Molecularly imprinted polymer nanomaterials and nanocomposites by controlled/living radical polymerization. Prog. Polym. Sci..

[177] BelBruno J.J. (2019). Molecularly imprinted polymers. Chem. Rev..

[178] Prasad B.B., Prasad A., Tiwari M.P., Madhuri R. (2013). Multiwalled carbon nanotubes bearing ’terminal monomeric unit’ for the fabrication of epinephrine imprinted polymer-based electrochemical sensor. Biosens. Bioelectron..

[179] Phonklam K., Wannapob R., Sriwimol W., Thavarungkul P., Phairatana T. (2020). A novel molecularly imprinted polymer PMB/MWCNTs sensor for highly-sensitive cardiac troponin T detection. Sens. Actuators B.

[180] Wang Y., Han M., Ye X., Wu K., Wu T., Li C. (2017). Voltammetric myoglobin sensor based on a glassy carbon electrode modified with a composite film consisting of carbon nanotubes and a molecularly imprinted polymerized ionic liquid. Microchim. Acta.

[181] Kangkamano T., Numnuam A., Limbut W., Kanatharana P., Thavarungkul P. (2017). Chitosan cryogel with embedded gold nanoparticles decorated multiwalled carbon nanotubes modified electrode for highly sensitive flow based non-enzymatic glucose sensor. Sens. Actuators B.

[182] Kumar T.H.V., Sundramoorthy A.K. (2018). Non-enzymatic electrochemical detection of urea on silver nanoparticles anchored nitrogen-doped single-walled carbon nanotube modified electrode. J. Electrochem. Soc..

[183] Messaoud N.B., Ghica M.E., Dridi C., Ali M.B., Brett C.M.A. (2017). Electrochemical sensor based on multiwalled carbon nanotube and gold nanoparticle modified electrode for the sensitive detection of bisphenol A. Sens. Actuators B.

[184] Zhang Y., Gao G., Liu H., Fu H., Fan J., Wang K., Chen Y., Li B., Zhang C., Zhi X. (2014). Idendification of volatile biomarkers of gastric cancer cells and ultrasensitive electrochemical detection based on sensing interface of Au-Ag alloy coated MWCNTs. Theranostics.

[185] Zhao Y., Qin J., Xu H., Gao S., Jiang T., Zhang S., Jin J. (2019). Gold nanorods decorated with graphene oxide and multi-walled carbon nanotubes for trace level voltammetric determination of ascorbic acid. Microchim. Acta.

[186] Asadian E., Shahrokhian S., Zad A.I., Ghorbani-Bidkorbeh F. (2017). Glassy carbon electrode modified with 3D graphene-carbon nanotube network for sensitive electrochemical determination of methotrexate. Sens. Actuators B.

[187] Huang B., Liu J., Lai L., Yu F., Ying X., Ye B.-C., Li Y. (2017). A free standing electrochemical sensor based on graphene foam-carbon nanotube composite coupled with gold nanoparticles and its sensing application for electrochemical determination of dopamine and uric acid. J. Electroanal. Chem..

[188] Qian Q., Hu Q., Li L., Shi P., Zhou J., Kong J., Zhang X., Sun G., Huang W. (2018). Sensitive fiber microelectrode made of nickel hydroxide nanosheets embedded in highly-aligned carbon nanotube scaffold for nonenzymatic glucose determination. Sens. Actuators B.

[189] Palve Y.P., Jha Y.P. (2020). A novel bilayer of copper nanowire and carbon nanotube electrode for highly sensitive enzyme free glucose detection. Mater. Chem. Phys..

[190] Tan C., Dutta G., Yin H., Siddiqui S., Arumugam P.U. (2018). Detection of neurochemicals with enhanced sensitivity and selectivity via hybrid multiwall carbon nanotube-ultrananocrystalline diamond microelectrodes. Sens. Actuators B.

[191] Zhang S., Zahed M.A., Sharifuzzaman M., Yoon S., Hui X., Barman S.C., Sharma S., Yoon S., Yoon H.S., Parl C. (2021). A wearable battery-free wireless and skin-interfaced microfluidics integrated electrochemical sensing patch for on-site biomarkers monitoring in human perspiration. Biosens. Bioelectron..

[192] Fan X., White I.M., Shopova S.I., Zhu H., Suter J.D., Sun Y. (2008). Sensitive optical biosensors for unlabeled targets: A review. Anal. Chim. Acta.

[193] Chen C., Wang J. (2020). Optical biosensors: An exhaustive and comprehensive review. Analyst.

[194] Li C., Shi G. (2014). Carbon nanotube-based fluorescence sensors. J. Photochem. Photobiol. C.

[195] Kim J.H., Ahn J.-H., Barone P.W., Jin H., Zhang J., Heller D.A., Strano M.S. (2010). A luciferase/single-walled carbon nanotube conjugate for near-infrared fluorescent detection of cellular ATP. Angew. Chem. Int. Ed..

[196] Heller D.A., Pratt G.W., Zhang J., Nair N., Hansborough A.J., Boghossian A.A., Reuel N.F., Barone P.W., Strano M.S. (2011). Peptide secondary structure modulates single-walled carbon nanotube fluorescence as a chaperone sensor for nitroaromatics. Proc. Natl. Acad. Sci. USA.

[197] Zhang J., Boghossian A.A., Barone P.W., Rwei A., Kim J.-H., Lin D., Heller D.A., Hilmer A.J., Nair N., Reuel N.F. (2011). Single molecule detection of nitric oxide enabled by d(AT)15 DNA adsorbed to near infrared fluorescent single-walled carbon nanotubes. J. Am. Chem. Soc..

[198] Zhang J., Kruss S., Hilmer A.J., Shimizu S., Schmois Z., De La Cruz F., Barone P.W., Reuel N.F., Heller D.A., Strano M.S. (2014). A rapid, direct, quantitative, and label-free detector of cardiac biomarker troponin T using near-infrared fluorescent single-walled carbon nanotube sensors. Adv. Healthc. Mater..

[199] Safee M.M., Gravely M., Roxbury D. (2021). A wearable optical microfibrous biomaterial with encapsulated nanosensors enables wireless monitoring of oxidative stress. Adv. Funct. Mater..

[200] Ma H., Xue N., Li Z., Xing K., Miao X. (2018). Ultrasensitive detection of miRNA-155 using multi-walled carbon nanotube-gold nanocomposites as a novel fluorescence quenching platform. Sens. Actuators B.

[201] Zhu W., Qin W., Atasoy U., Sauter E. (2009). Circulating microRNAs in breast cancer and healthy subjects. BMC Res. Notes.

[202] Mattiske S., Suetani R.J., Neilsen P.M., Callen D.F. (2012). The oncogenic role of miR-155 in breast cancer. Cancer Epidemiol. Biomarkers Prev..

[203] Elmizadeh H., Faridbod F., Soleimani M., Ganjali M.R., Bardajee G.R. (2020). Fluorescent apta-nanobiosensors for fast and sensitive detection of digoxin in biological fluids using rGQDs: Comparison of two approaches for immobilization of apatamer. Sens. Actuators B.

[204] Wang X., Wang C., Qu K., Song Y., Ren J., Miyoshi D., Sugimoto N., Qu X. (2010). Ultrasensitive detection of a prognostic indicator in early-stage cancer using graphene oxide and carbon nanotubes. Adv. Funct. Mater..

[205] Prabowo B.A., Purwidyantri A., Liu K.-C. (2018). Surface plasmon resonance optical sensor: A review on light source technology. Biosens..

[206] Lisi S., Scaranoa S., Fedelia S., Pascalea E., Cicchia S., Raveletb C., Peyrinb E., Minunni M. (2017). Toward sensitive immuno-based detection of tau protein by surface plasmon resonance coupled to carbon nanostructures as signal amplifiers. Biosens. Bioelectron..

[207] Qiu W., Xu H., Takalkar S., Gurung A.S., Liu B., Zheng Y., Guo Z., Baloda M., Baryeh K., Liu G. (2015). Carbon nanotube-based lateral flow biosensor for sensitive and rapid detection of DNA sequence. Biosens. Bioelectron..

[208] Yao L.T.J., Zhu M.Z.L., Zhong Y., Liu G., Xue F., Chen W. (2016). MWCNTs based high sensitive lateral flow strip biosensor for rapid determination of aqueous mercury ions. Biosens. Bioelectron..

[209] Nandeshwar R., Tallur S. (2021). Integrated low cost optical biosensor for high resolution sensing of myeloperoxidase (MPO) activity through carbon nanotube degradation. IEEE Sens. J..

[210] Pinals R.L., Ledesma F., Yang D., Navarro N., Jeong S., Pak J.E., Kuo L., Chuang Y.-C., Cheng Y.-W., Sun H.-Y. (2021). Rapid SARS-CoV-2 spike protein detection by carbon nanotube-based near-infrared nanosensors. Nano Lett..

[211] Heller D.A., Balik S., Eurell T.E., Strano M.S. (2005). Single-walled carbon nanotube spectroscopy in live cells: Towards long-term labels and optical sensors. Adv. Mater..

[212] Chen Z., Tabakman S.M., Goodwin A.P., Kattah M.G., Daranciang D., Wang X., Zhang G., Li X., Liu Z., Utz P.J. (2008). Protein microarrays with carbon nanotubes as multicolor Raman labels. Nat. Biotechnol..

[213] Bajaj P., Mikoryak C., Wang R., Bushdiecker D.K., Memon P., Draper R.K., Dieckmann G.R., Pantano P., Musselman I.H. (2014). A carbon nanotube-based Raman-imaging immunoassay for evaluating tumor targeting ligands. Analyst.

[214] Chen Y.C., Young R.J., Macpherson J.V., Wilson N.R. (2011). Silver-decorated carbon nanotube networks as SERS substrates. J. Raman Spectrosc..

[215] Sinha S.S., Jones S., Pramanik A., Ray P.R. (2016). Nanoarchitecture based SERS for biomolecular fingerprinting and label-free disease markers diagnosis. Acc. Chem. Res..

[216] Tran T.-T., Mulchandani A. (2016). Carbon nanotubes and graphene nano field-effect transistor-based biosensors. Trends Anal. Chem..

[217] Sireesha M., Babu V.J., Kiran A.S.K., Ramakrishna S. (2018). A review on carbon nanotubes in biosensor devices and their applications in medicine. Nanocomposites.

[218] Cao Q., Tersoff J., Farmer D.B., Zhu Y., Han S.-J. (2017). Carbon nanotube transistors scaled to a 40-nanometer footprint. Science.

[219] Tlili C., Myung N.V., Shetty V., Mulchandani A. (2011). Label-free, chemiresistor immunosensor for stress biomarker cortisol in saliva. Biosens. Bioelectron..

[220] Garcia-Aljaro C., Cella L.N., Shirale D.J., Park M., Munoz F.J., Yates M.V., Mulchandani A. (2010). Carbon nanotubes-based chemiresistive biosensors for detection of microorganisms. Biosens. Bioelectron..

[221] Martinez M.T., Tseng Y.-C., Ormategui N., Loinaz I., Eritja R., Bokor J. (2009). Label-free DNA biosensors based on functionalized carbon nanotube field effect transistor. Nano Lett..

[222] Kim J.P., Lee B.Y., Lee J., Hong S., Sim S.J. (2009). Enhancement of sensitivity and specificity by surface modification of carbon nanotubes in diagnosis of prostate cancer based on carbon nanotube field effect transistors. Biosens. Bioelectron..

[223] Oh J., Yoo G., Chang Y.W., Kim H.J., Jose J., Kim E., Pyun J.-C., Yoo K.-H. (2013). A carbon nanotube metal semiconductor field effect transistor-based biosensor for detection of amyloid-beta in human serum. Biosens. Bioelectron..

[224] Ramnani P., Gao Y., Ozsoz M., Mulchandani A. (2013). Electronic detection of microRNA at attomolar level with high specificity. Anal. Chem..

[225] Islam A.E. (2013). Variability and reliability of single-walled carbon nanotube field effect transistors. Electronics.

[226] Rajesh Sharma V., Puri N.K., Singh R.K., Biradar A.M., Mulchandani A. (2013). Label-free detection of cardiac troponin-I using gold nanoparticles functionalized single-walled carbon nanotubes based chemiresistive biosensor. Appl. Phys. Lett..

[227] Puri N., Niazi A., Biradar A.M., Mulchandani A. (2014). Conducting polymer functionalized single-walled carbon nanotube based chemiresistive biosensor for the detection of human cardiac myoglobin. Appl. Phys. Lett..

[228] Tan F., Saucedo N.M., Ramnani P., Mulchandani A. (2015). Label-free electrical immunosensor for highly sensitive and specific detection of microcystin-LR in water samples. Environ. Sci. Technol..

[229] Hwang S.I., Franconi N.G., Rothfuss M.A., Bocan K.N., Bian L., White D.L., Burkert S.C., Euler R.W., Sopher B.J., Vinay M.L. (2019). Tetrahydrocannabinol detection using semiconductor-enriched single-walled carbon nanotube chemiresistors. ACS Sens..

[230] Wasik D., Mulchandani A., Yates M.V. (2017). A heparin-functionalized carbon nanotube-based affinity biosensor for dengue virus. Biosens. Bioelectron..

[231] Etheridge A., Lee I., Hood L., Galas D., Wang K. (2011). Extracellular microRNA: A new source of biomarkers. Mutat. Res..

[232] Cella L.N., Sanchez P., Zhong W., Myung N.V., Chen W., Mulchandani A. (2010). Nano aptasensor for protective antigen toxin of anthrax. Anal. Chem..

[233] Das B.K., Tlili C., Badhulika S., Lakshmi N.C., Chen W., Mulchandani A. (2011). Single-walled carbon nanotubes chemiresistor aptasensors for small molecules: Picomolar level detection of adenosine triphosphate. Chem. Commun..

[234] Tanaka Y., Oda S., Yamaguchi H., Kondo Y., Kojima C., Ono A. (2007). 15N–15N J-coupling across HgII: Direct observation of HgII-mediated T-T base pairs in a DNA duplex. J. Am. Chem. Soc..

[235] Gong J.-L., Sarkar T., Badhulika S., Mulchandani A. (2013). Label-free chemiresistive biosensor for mercury (II) based on single-walled carbon nanotubes and structure switching DNA. Appl. Phys. Lett..

[236] Shao W., Shurin M.R., Wheeler S.E., He X., Star A. (2021). Rapid detection of SARS-CoV-2 antigens using high-purity semiconducting single-walled carbon nanotube-based field-effect transistors. ACS Appl. Mater. Interfaces.

[237] Liang Y., Xiao M., Wu D., Lin Y., Liu L., He J., Zhang G., Peng L.-M., Zhang Z. (2020). Wafer-scale uniform carbon nanotube transistors for ultrasensitive and label-free detection of disease biomarkers. ACS Nano.

[238] Kang T.-H., Lee S.-W., Hwang K., Shim W., Lee K.-Y., Lim J.-A., Yu W.-R., Choi I.-S., Yi H. (2020). All-inkjet-printed flexible nanobio-devices with efficient electrochemical coupling using amphiphilic biomaterials. ACS Appl. Mater. Interfaces.

[239] Lee K.-Y., Byeon H.-H., Jang C., Choi J.-H., Choi I.-S., Jung Y., Kim W., Chang J., Yi H. (2015). Hydrodynamic assembly of conductive nanomesh of single-walled carbon nanotubes using biological glue. Adv. Mater..

[240] Shen Y., Modha S., Tsutsui H., Mulchandani A. (2021). An origami electrical biosensor for multiplexed analyte detection in body fluids. Biosens. Bioelectron..

[241] Eatemadi A., Daraee H., Karimkhanloo H., Kouhi M., Zarghami N., Akbarzadeh A., Abasi M., Hanifehpour Y., Joo S.W. (2014). Carbon nanotubes: Properties, synthesis, purification, and medical applications. Nanoscale Res. Lett..

[242] Tang J., Cao Q., Tulevski G., Jenkins K.A., Nela L., Farmer D.B., Han S.-J. (2018). Flexible CMOS integrated circuits based on carbon nanotubes with sub-10 ns stage delays. Nat. Electron..

[243] Zhang H., Xiang L., Yang Y., Xiao M., Han J., Ding L., Zhang Z., Hu Y., Peng L.-M. (2018). High-performance carbon nanotube complementary electronics and integrated sensor systems on ultrathin plastic foil. ACS Nano.

[244] Morales-Narvaez E., Dincer C. (2020). The impact of biosensing in a pandemic outbreak: COVID-19. Biosens. Bioelectron..

[245] Vermisoglou E., Panacek D., KJayaramulu D., Pykal M., Frebort I., Kolar M., Hajduch M., Zboril R., Otyepka M. (2020). Human virus detection with graphene-based maerials. Biosens. Bioelectron..

